# Current status and advances in esophageal drug delivery technology: influence of physiological, pathophysiological and pharmaceutical factors

**DOI:** 10.1080/10717544.2023.2219423

**Published:** 2023-06-21

**Authors:** Ai Wei Lim, Nicholas J. Talley, Marjorie M. Walker, Gert Storm, Susan Hua

**Affiliations:** a Therapeutic Targeting Research Group, School of Biomedical Sciences and Pharmacy, University of Newcastle, Callaghan, NSW, Australia; b Precision Medicine Research Program, Hunter Medical Research Institute, New Lambton Heights, NSW, Australia; c School of Medicine and Public Health, University of Newcastle, Callaghan, NSW, Australia; d Department of Pharmaceutics, Utrecht Institute for Pharmaceutical Sciences, Utrecht University, Utrecht, The Netherlands; e Department of Biomaterials Science and Technology, MIRA Institute for Biomedical Technology and Technical Medicine, University of Twente, Enschede, The Netherlands; f Department of Surgery, Yong Loo Lin School of Medicine, National University of Singapore, Singapore

**Keywords:** Esophagus, esophageal drug delivery, topical, gastrointestinal, conventional formulations, orodispersible tablets, stents, films, nanoparticles, translation

## Abstract

Diseases affecting the esophagus are common. However, targeted drug delivery to the esophagus is challenging due to the anatomy and physiology of this organ. Current pharmacological treatment for esophageal diseases predominantly relies on the off-label use of drugs in various dosage forms, including those for systemic drug delivery (e.g. oral tablets, sublingual tablets, and injections) and topical drug delivery (e.g. metered dose inhaler, viscous solution or suspension, and endoscopic injection into the esophagus). In general, systemic therapy has shown the most efficacy but requires the use of high drug doses to achieve effective concentrations in the esophagus, which increases the risk of adverse effects and toxicity. Topical drug delivery has enormous potential in improving the way we treat patients with acute and chronic esophageal diseases, especially those requiring drugs that have low therapeutic index and/or significant adverse effects to non-targeted organs and tissues. This review will address the physiological, pathophysiological, and pharmaceutical considerations influencing topical drug delivery in the esophagus. The main conventional (e.g. liquid formulations, orodispersible tablets, lozenges, pastilles, troches, chewing gum) and innovative (e.g. stent-based, film-based, nanoparticulate-based) drug delivery approaches will be comprehensively discussed, along with the developments to improve their effectiveness for topical esophageal drug delivery. The translational challenges and future clinical advances of this research will also be discussed.

## Introduction

Diseases affecting the esophagus are common. These include gastroesophageal reflux disease (GERD), achalasia, infections, eosinophilic esophagus (EoE), Barrett’s esophagus, and esophageal cancer. These disease states correspond with alteration in the physiology of the organ, with some sharing similar symptomatic and clinical presentations but vary significantly in their etiology and therapeutic management. These pathologies include neuromuscular dysfunction, inflammation, infection, and neoplasms of the esophagus. Owing to the anatomy and physiology of the esophagus, targeted drug delivery to this organ is a significant challenge, as it serves as an effective barrier against the external environment. Current pharmacological treatment for esophageal diseases predominantly relies on the off-label use of drugs in various dosage forms, including those for systemic drug delivery (e.g. oral tablets, sublingual tablets, and injections) and topical drug delivery (e.g. metered dose inhaler, viscous solution or suspension, and endoscopic injection into the esophagus). In general, systemic therapy has shown the most efficacy but requires the use of high drug doses to achieve effective concentrations in the esophagus, as the local blood supply to this organ is relatively poor (Zhang et al., 2008). This mode of delivery significantly increases the risk of adverse effects and toxicity in non-target organs, especially at the high doses and/or long-term dosing regimens required.

Although topical drug delivery has obvious advantages for the treatment of esophageal diseases, there are currently very few marketed products available that are specifically indicated for the localized treatment of esophageal diseases. Local delivery of drugs across the esophageal mucosa is difficult due to the biological barriers that makes this organ relatively impermeable to compounds. Nevertheless, there is an urgent need for improved treatments for esophageal diseases that are both effective and safe. Further development and optimization of esophageal drug formulations have led to improvements in drug availability and formulation retention. However, despite the pharmaceutical advances in esophageal drug delivery to date, very few of them have translated to the clinical phase. This review will address the physiological, pathophysiological, and pharmaceutical considerations influencing esophageal drug delivery and formulation approaches. The translational challenges and development aspects of novel formulations will also be discussed.

## Physiological factors influencing esophageal drug delivery

The esophagus is a part of the gastrointestinal tract (GI tract) that connects the pharynx to the stomach. It is a hollow, muscular channel that delivers swallowed food bolus to the stomach. The thickness of the esophageal wall in healthy individuals varies depending on the section of the esophagus, with the largest wall thickness during esophageal contraction of 4.70 mm (95%CI: 4.44-4.95) and during esophageal dilation of 2.11 mm (95%CI: 2.00-2.23) (Xia et al., 2009). The esophagus begins at the upper esophageal sphincter that is formed by the cricopharyngeal muscle and ends with the lower esophageal sphincter, which is surrounded by the crural diaphragm (Standring, 2020). While the average length of the esophagus in an adult is between 23 to 25 cm, the length in children at birth varies between 8 to 10 cm (Standring, 2020; Scott-Brown et al., 2008). The esophagus is lined with non-keratinized squamous epithelium in humans and the muscular elements are smooth muscle (Standring, 2020).

Despite the constant exposure of the esophageal lining to friction and irritants from food boluses, pathogens, food antigens, and acidic stomach contents, the esophagus remains uninjured under normal circumstances owing to the innate defense mechanisms. These defense mechanisms involve the clearance of luminal content and epithelial resistance contributed by the physical barrier of the esophagus (Sarosiek & McCallum, 2000). Esophageal clearance is achieved via peristalsis with the aid of saliva, whilst epithelial resistance involves multiple levels of support from intracellular and extracellular components (Sarosiek & McCallum, 2000). While these mechanisms protect the esophagus from the luminal contents, they can pose significant challenges to effective topical drug delivery, which will be discussed in the following section.

### Transit time

Peristalsis is the main mechanism that the esophagus uses to carry out its function. It is also one of the organ’s defence mechanisms to clear acidic refluxates from the lumen. In healthy states, esophageal transit time can vary from seconds up to two minutes depending on the characteristics of the contents (e.g. nature, size, and water content), body posture, and general physiology (Osmanoglou et al., 2004; Cordova-Fraga et al., 2008). The speed of the peristaltic waves has been reported to be between 2 to 6 cm per second (Batchelor, 2005). The esophagus transit time for solid/semi-solid food and liquids is usually between 4 to 8 seconds and 1 to 2 seconds, respectively (Osmanoglou et al., 2004; Cordova-Fraga et al., 2008). With the influence of gravitational force, the transit time through the esophagus can vary with different body postures. Cordova et al. reported that the esophageal transit time is shortest in the Fowler position (45°), followed by upright (90°) and then the supine positions (Cordova-Fraga et al., 2008). Similar results were observed by Osmanoglou et al, which also highlighted that the amount of liquid taken with the bolus influenced the transit time (Osmanoglou et al., 2004). With regard to medications, longer mucosal contact time of drugs in the esophagus has been shown to have a positive correlation with disease improvement (Batchelor, 2005; Casiraghi et al., 2020). However, increasing the transit time of pharmaceutical formulations in the esophagus is challenging. The formulation should have sufficient mucosal retention to allow drug uptake across the mucosal barrier, without causing discomfort or irritation.

### Esophageal pH

The normal pH of the esophagus is approximately pH 7.0 (Tutuian & Castell, 2006). Esophageal pH can be affected in pathological diseases such as GERD and Barrett’s esophagus – whereby reflux of gastric acid and pepsin into the esophagus can reduce the luminal pH (≤ pH 4.0), leading to the associated signs and symptoms (Tutuian & Castell, 2006). While the stratified squamous epithelium of the esophagus is equipped with defence mechanisms, acids can pass through the epithelium when the pH level falls below 2.0 and the exposure time is prolonged (Sarosiek & McCallum, 2000). For example, Dvorak et al. reported that pH <2.0 is common in Barrett’s esophagus, and the acid exposure time and frequency are significantly higher in this condition (28.8 ± 3.6 seconds and 79 ± 11.4 episodes, respectively) compared to GERD (15.6 ± 1.2 seconds and 48.3 ± 8.8 episodes, respectively) (Dvorak et al., 2006; Tutuian & Castell, 2006).

Acids in the esophagus stimulate the activation of the esophagus-salivary reflex to enhance bicarbonate, mucus, and saliva secretion to neutralize the esophageal pH (Helm et al., 1987; Dutta et al., 1992; Kongara & Soffer, 1999). Interestingly, Campisi et al. demonstrated that the pH of the saliva produced in GERD patients (pH 8.9) was higher in comparison to healthy individuals (pH 7.9) (Campisi et al., 2008). Dutta et al. (1992) reported that there was no increase in saliva flow rate following exposure of the esophagus to hydrochloric acid (HCl) solution in healthy individuals at a concentration of 10 mmol/L (pH 2.2) and 15 mmol/L (pH 2.0) for up to 30 minutes; however, the saliva flow rate increased when the duration of exposure was above 60 minutes. When the HCl concentration was significantly increased to 50 mmol/L with pH 1.8, an increase in saliva flow rate (4 to 7-fold) and bicarbonate secretion in saliva (21 to 43-fold) was observed. Conversely, Campisi et al. reported using a similar study design that the stimulated flow rate of saliva in GERD patients was lower (0.99 mL/min) compared to healthy individuals (1.2 mL/min) (Campisi et al., 2008) – this finding is also consistent with the study conducted by Namiot et al. (1994). Therefore, local pH needs to be considered for topical drug delivery in the esophagus as it can affect the solubility and ionization state of drugs, which will ultimately affect drug absorption into the esophageal mucosal tissue.

### Mucus

The esophageal mucus is thought to act as a buffer layer on the surface of the mucosa to neutralize and protect the esophagus from stomach refluxates. It also plays a role in the innate immune system that forms a barrier against pathogens (Sarosiek & McCallum, 2000; Nochi & Kiyono, 2006). Mucus is produced in the esophagus mainly by the esophageal submucosal glands. These glands are connected to the lumen of the esophagus via small ducts that are located between the submucosa and mucosa of the epithelium (Meyer et al., 1986). Esophageal mucus contains a mixture of mucin, proteins (e.g. threonine 16.3%, serine 14.2%, glycine 8.9%, glutamine 8.5%, alanine 8.4%, proline 8.0%, asparagine 7.6%, leucine 6.9%, valine 5.7%, lysine 3.3%, isoleucine 2.8%, histidine 2.6%, arginine 2.6%, phenylalanine 2.6%, and tyrosine 1.1% (Namiot et al., 1994)), polypeptides (e.g. epidermal growth factors, prostaglandin E_2_, and immunoglobulin A (Sarosiek et al., 1993, 1994)), phospholipids, and bicarbonate ions (Namiot et al., 1994).

Although the mucosa throughout the GI tract is overlayed with a mucus layer, the thickness and composition vary depending on the organ. For example, the esophagus and small intestine have only one type of mucus that is unattached and loose (Atuma et al., 2001; Hansson, 2012). In contrast, the stomach and colon have a two-layered mucus system comprising of an inner, attached mucus layer and an outer mucus layer that is unattached and loose (Hansson, 2012). Compared to other regions of the GI tract, the esophageal mucus layer is relatively thin at an estimated 30 μm (Taherali et al., 2018). This unattached mucus may contribute to rapid drug clearance from the esophagus following topical administration. It should be noted that the mucus layer covering the human esophageal mucosa can differ depending on the pathophysiological state of the esophagus. For example, Dixon et al. reported a complete absence of adherent mucus layer on normal human esophagus and a significant adherent mucus layer (containing neutral and acidic mucins) in esophageal biopsies from patients with columnar-lined Barrett’s esophagus (Dixon et al., 2001).

Mucus with higher viscosity could negatively affect the topical penetration of drugs across the esophageal mucosa (Taherali et al., 2018). The viscosity of the mucus is approximately 130 cP, but increases when the esophagus is exposed to acid and pepsin – likely due to an increased secretion of phospholipids into the mucus that may offer protection from luminal injury (Namiot et al., 1994). However, there are studies that suggests that the relatively low volume of mucus produced in the esophagus has little to no role in protecting the esophageal mucosa (Dixon et al., 2001; Orlando, 2010; Taherali et al., 2018). For example, the number of mucus glands in the esophagus has been reported to be limited and are only able produce soluble mucus that lack sufficient viscoelasticity to form a stable lining on the surface of the mucosa (Dixon et al., 2001; Orlando, 2010).

The barrier role of the esophageal mucus for topical drug delivery has not been comprehensively investigated. It is likely that the physical characteristic of the mucus layer could potentially impede the penetration of drugs across the esophageal mucosa and should be considered in topical formulation design. Understanding the interactions of the esophageal mucus with drug molecules as well as drug delivery systems will help to optimize topical drug formulations for clinical use.

### Saliva

Swallowed saliva produced in the oral cavity contributes to the mucus lining on the luminal surface of the esophagus. Human saliva is composed of 97 to 99% water accompanied by mucins, electrolytes, proteins, lipids, enzymes, growth factors, and inflammatory mediators (Dawes et al., 2015). Electrolytes such as sodium, potassium, chloride, calcium, phosphate, and bicarbonate contribute to the ionic strength of the fluid (Almståhl & Wikström, 2003). Other components in the saliva includes phospholipids, cholesterol, free fatty acids, glycerin, and triglycerides (Almståhl & Wikström, 2003). Aside from lubricating the lumen of the esophagus, saliva also acts as a buffer to neutralize refluxates from the stomach and aids to restore esophageal pH (Dawes et al., 2015). During the unstimulated state, saliva has a low bicarbonate concentration that is around 5.0 mmol/L, thereby conferring only weak buffering capacity (Dawes et al., 2015; Dosedělová et al., 2020). However, the bicarbonate concentration in saliva increases significantly (ranging between 8.0 to 24.0 mmol/L) during the stimulated state or disease state (e.g. GERD) (Bardow et al., 2000; Dosedělová et al., 2020). Therefore, composition of the saliva may affect topical drug absorption across the esophageal mucosa by altering the physicochemical properties of certain drugs or drug delivery systems, which can lead to factors such as agglomeration and poor drug solubility.

Saliva production in a healthy individual is about 0.5 to 1.5 L a day (Iorgulescu, 2009). The flow rate of saliva in the oral cavity varies depending on the physiological state of an individual. In the unstimulated state, the flow rate of saliva is about 0.3 to 0.4 mL/min and this increases to 4 to 5 mL/min in the stimulated state (e.g. eating and chewing) (Iorgulescu, 2009). During the resting state or sleep, the flow rate of saliva decreases significantly to about 0.1 to 0.25 mL/min (Iorgulescu, 2009). The swallowing reflex is usually triggered when the volume of saliva in the oral cavity reaches about 1.1 mL, which causes the saliva to wash over the mucosa during transit through the esophagus (Iorgulescu, 2009). The number of swallows a person makes when awake is about 20 to 350 times an hour compared to approximately 3 times per hour when asleep (Sato & Nakashima, 2006). Therefore, the continuous washing of saliva through the esophagus is a significant challenge for effective topical drug delivery. Drugs formulations will need to have sufficient retention to the esophageal mucosal surface to avoid being washed down the GI tract (Batchelor et al., 2004).

### Esophageal epithelial barrier

**Figure 1.**
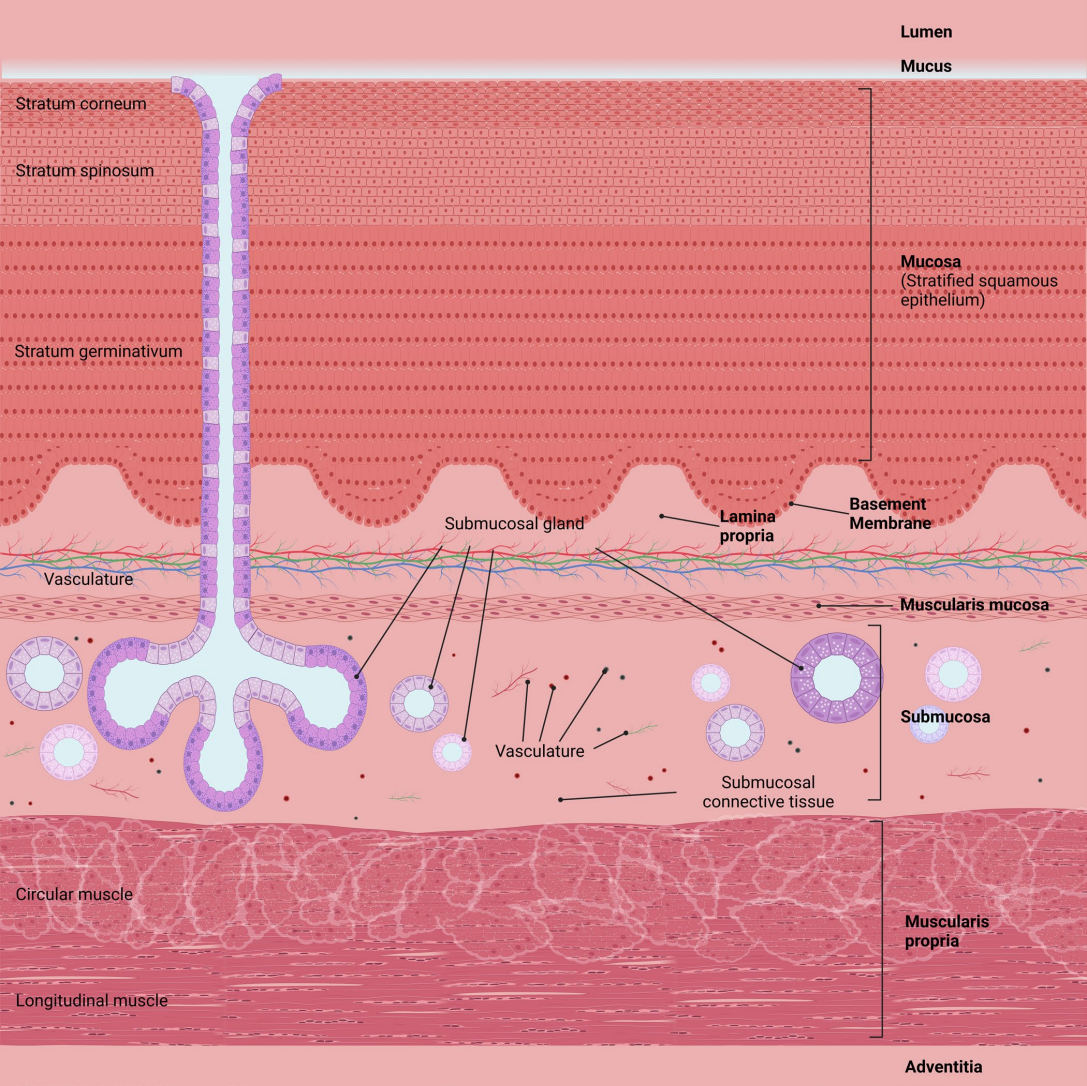
Schematic diagram of the esophageal lining (cross-section).

The average thickness of the esophageal wall is approximately 1.87 to 2.70 mm in the dilated state and 4.05 to 5.68 mm in the contracted state (Xia et al., 2009). The thickness of the esophageal wall has also been reported to be slightly larger in males (5.26 mm) compared to females (4.34 mm) (Xia et al., 2009). The wall of the esophagus is comprised of the mucosa, submucosa, and muscularis propria (Figure 1). In healthy individuals, the mucosa is composed of three layers – non-keratinized, stratified squamous epithelium; lamina propria (composed of connective tissue); and muscularis mucosa (Scott-Brown et al., 2008; Orlando, 2010; Standring, 2020). The muscularis mucosa is composed primarily of smooth muscle, with a combination of striated muscles at the upper part of the esophagus. The submucosa layer consists of predominantly blood vessels, lymphatic vessels, minor salivary glands, connective tissues, and autonomic nerve plexus (i.e. submucosal plexus). The muscularis propria is formed by a mixture of striated and smooth muscles and is responsible for motor functions of the esophagus.

**Figure 2.**
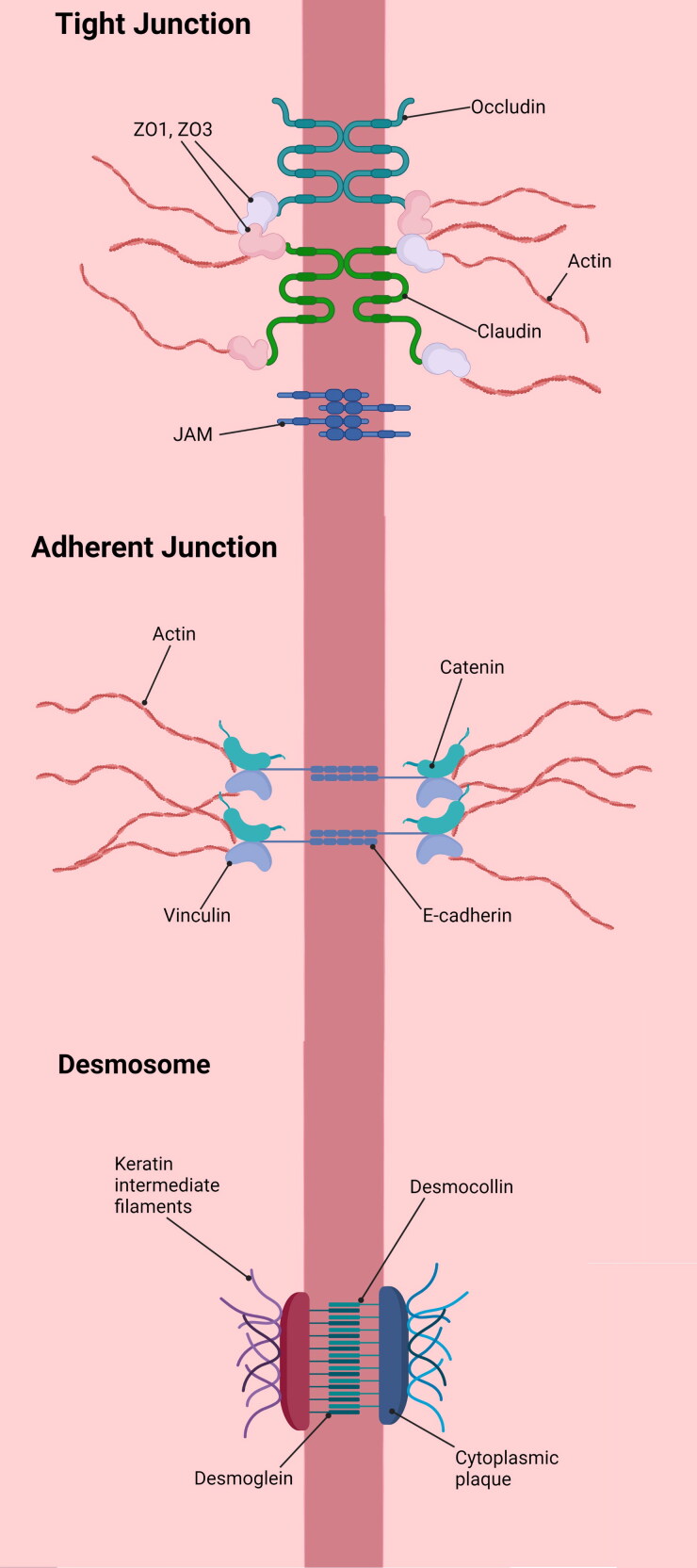
Schematic diagram of the apical junction complex.

The presence of stratified squamous epithelium is exclusive to the oral and esophageal mucosa in the GI tract of humans (Terashi et al., 2000). This layer is approximately 30 cells thick (Orlando, 2010). It is composed of three functional layers, namely the stratum corneum, stratum spinosum and stratum germinativum (Wang, 2017; Stanforth et al., 2022). The stratum corneum is approximately 7 to 8 cells thick and is comprised of flat stratified squamous cells that are bound with filaggrin and surrounded by glycocalyx to forms a tight protective barrier against luminal contents in the esophagus (Su et al., 2020). The stratum spinosum consists of cells that are linked by desmosomes, which are strong intercellular junctions that provide resistance to the cells toward mechanical stress (Su et al., 2020). The stratum germinativum is the basal cell layer, containing cells that proliferate and move toward the surface of the epithelium to replace lost cells (Su et al., 2020). Cell proliferation in the stratum germinativum is initiated within 30 minutes after the esophagus is exposed to acid (Orlando, 2010). New cells take around 7 to 8 days to migrate from the stratum germinativum to the surface of the esophageal lumen (Chandrasoma, 2018). Cells within the stratum corneum and stratum spinosum are connected to one another by tight junctions and adherent junctions, forming the apical cell membrane and junction complex (Figure 2) (Su et al., 2020). This complex serves to regulate paracellular transit of ions and molecules as well as cell-cell signaling (Deli, 2009). The apical cell membrane is a lipid bilayer that is highly hydrophobic in nature, thus is difficult for acids to pass through (Orlando, 2010). The apical cell membrane and junction complex forms a strong physical barrier that serves as one of the defence mechanisms to protect the esophageal lining from injury caused by mechanical stress during peristalsis and the exposure to acidic stomach refluxates (Orlando, 2010).

For topical drug delivery, the esophageal epithelial barrier can make it difficult for drugs to penetrate. However, this barrier function has been shown to be altered in esophageal disease states (Blevins et al., 2018). For example, dilation of the intercellular space, loss of tight junction protein, abnormal adherens junction complex, and spongiosis have been observed in EoE, GERD, and Barrett’s esophagus (de Hertogh et al., 2006; Mueller et al., 2006; Vieth et al., 2016; Blevins et al., 2018). These changes increase paracellular permeability, which is likely to enhance drug absorption across the esophageal mucosa (Deli, 2009; Hashimoto et al., 2019; Brunner et al., 2021). However, it should be noted that the reduced barrier function may also increase the vulnerability of the esophageal mucosa to luminal contents, including pathogens and irritants (Blevins et al., 2018).

## Conventional pharmaceutical approaches for topical drug delivery to the esophagus

Topical drug delivery to the esophagus is challenging with conventional pharmaceutical approaches. Conventional esophageal dosage forms can be categorized into two groups – liquid dosage forms (e.g. viscous oral liquids) and solid dosage forms (e.g. orodispersible tablets, lozenges, chewing gums) (Table 1). For effective esophageal drug delivery, rational formulation design should consider the (i) retention time of the formulation to the esophageal mucosa, (ii) dissolution rate of the formulation, (iii) rate of drug absorption into the mucosa, (iv) extent of esophageal coverage, (v) and degree of systemic bioavailability of the drugs. Ideally, topical formulations to treat pathological conditions that are restricted to the esophagus should have maximal accumulation in the esophagus and minimal systemic exposure. The formulation should also be convenient for self-administration, easy to swallow, and palatable to ensure compliance. This section will discuss the main conventional esophageal dosage forms and the developments to improve their effectiveness for topical esophageal drug delivery. It should be noted that endoscopic injection of conventional drug solutions or suspensions directly into the esophagus has been used clinically (Arora et al., 2017; Tascone & Halbert, 2019); however, this is beyond the scope of this review paper.

**Table 1.** Summary of the advantages and limitations of conventional and innovative pharmaceutical approaches for topical drug delivery to the esophagus.

Drug delivery strategy		Advantages	Limitations	References
**Conventional liquid formulations**	**Sucralfate**	Viscous Mucoadhesive Able to bind specifically to injured mucosal tissue to provide a physical barrier against irritants Generally well tolerated Low absorption throughout GI tract May increase viscosity of mucus	Drug delivery using sucralfate as a mucoadhesive base has not been explored May impede drug absorption across mucosa May reduce the bioavailability of other co-administered drugs	Nagashima, 1981; Brogden et al., 1984; Slomiany et al., 1986; Orlando et al., 1987; Tytgat, 1987; Jørgensen & Elsborg, 1991; Sulochana et al., 2016; Savarino et al., 2017a
	**Alginate**	Mucoadhesive Biocompatible and nontoxic Able to form hydrogels May provide protection to esophageal mucosa by forming a physical barrier	Further studies are required to evaluate the use of alginates for esophageal drug delivery	(Otterlei et al., 1991; Batchelor et al., 2002; Richardson et al., 2004; Lee & Lee, 2009; Batchelor, 2005; Lee & Mooney, 2012; Woodland et al., 2015)
	**Poloxamer 407**	Thermoreversible properties (liquid to gel) Mucoadhesive Biocompatible and inactive May provide protection to esophageal mucosa by forming a physical barrier May provide sustained release of drugs	Although it has shown promising results for mucosal drug delivery, there are limited studies focusing on its application for esophageal drug delivery May be used in combination with other mucoadhesive compounds, but this has not yet been explored in detail for esophageal drug delivery	(Dumortier et al., 2006; Ricci et al., 2002, 2005; Di Simone et al., 2012; Fakhari et al., 2017; Savarino et al., 2017b; Giuliano et al., 2018; Antonino et al., 2019)
	**Chitosan**	Mucoadhesive Biocompatible and nontoxic Exhibits antimicrobial property May enhance mucosal penetration of compounds by loosening epithelial tight junctions Capable of covering the entire surface of the esophagus following administration in clinical studies Extensive studies supporting its use in drug delivery, including for gastrointestinal drug delivery	Similar to other mucoadhesive compounds, there are limited studies focusing on its application for esophageal drug delivery	(Kotzé et al., 1998; Senel et al., 2000; Smith et al., 2004; Casettari et al., 2010; Martău et al., 2019)
	**Carbomer**	Mucoadhesive Generally considered safe and nontoxic Already used in various formulations as a thickener, stabilizer, and gelling agent Combination of carbomers have been used to formulate solid, semi-solid and liquid dosage forms	Further studies are required to assess its application for esophageal drug delivery (e.g. reproducibility in esophageal coverage, mucosal retention, and drug absorption) Lack of studies comparing carbomers with other mucoadhesive polymers for esophageal drug delivery	(Riley et al., 2001; Smart et al., 2003; Bonacucina et al., 2004; Kulkarni & Shaw, 2016; Brady et al., 2017; Mastropietro et al., 2017)
	**Xanthan gum**	Soluble in water to form a viscous solution with high pseudoplasticity Stable across wide range of parameters, including temperature and pH Generally considered safe for human consumption Already used as a thickening agent in liquid pharmaceutical formulations for patients with dysphagia	Has weak mucoadhesive properties, which may be improved via chemical modification Associated with increased risk of developing necrotizing enterocolitis in infants, especially those born prematurely Limited studies have investigated the combination of xanthan gum with other natural polymers for esophageal drug delivery	(García-Ochoa et al., 2000; Casiraghi et al., 2020; BeMiller, 2019; Patel et al., 2020; Sworn, 2021)
	**Thiolated hydroxypropyl-β-cyclodextrin**	Mucoadhesive	There are limited studies focusing on its application for esophageal drug delivery Further safety and toxicity studies are required	(Perrone et al., 2017; Laquintana et al., 2019)
**Other conventional dosage forms**	**Orodispersible tablets**	Do not require water for administration Fast disintegration in the oral cavity Shown promising results for topical drug delivery in eosinophilic oesophagitis (EoE)	Relatively new conventional formulation approach for esophageal drug delivery that requires further evaluation (e.g. mucosal retention time, degree of esophageal coverage, and depth of mucosal drug penetration) Relies on the innate mucoadhesive property of saliva to slow esophageal transit for drug absorption, which may not be as effective compared to exogenous mucoadhesive excipients Potential of systemic drug exposure for drugs that do not undergo high first pass metabolism	(Bandari et al., 2008; Ghosh et al., 2011; Dilger et al., 2013; Al-Khattawi & Mohammed, 2013; Miehlke et al., 2016; Lucendo et al., 2019; Miehlke et al., 2020; Straumann et al., 2020; Miehlke et al., 2022)
	**Lozenges, pastilles, and troches**	Do not require water for administration Retained within the oral cavity for gradual disintegration, dissolution, and drug release in saliva	Very few studies available that have investigated these dosage forms for esophageal drug delivery Relies on the innate mucoadhesive property of saliva to slow esophageal transit for drug absorption, which may not be as effective compared to exogenous mucoadhesive excipients Slow disintegration and dissolution in saliva May dilute drug concentration in saliva due to long retention time in oral cavity Potential for systemic absorption via sublingual and buccal regions Inconsistent drug release profile based on administration technique and hydration status of the oral cavity Not all drugs are suitable to be delivered via these dosage forms due to their physicochemical and/or pharmacodynamic properties Some drugs could cause staining in the oral cavity, be unpalatable, or cause local adverse effects (e.g. mucosal irritation)	(Zhang et al., 2002; Valenzano et al., 2014)
	**Chewing gum**	Does not require water for administration Active compound is gradually released and mixed with saliva as the gum is chewed Easy termination of drug release by removing the gum	Limited studies available that have investigated this dosage form for esophageal drug delivery Drug release can be inconsistent depending on chewing technique and release of active compound from the gum base Relies on the innate mucoadhesive property of saliva to slow esophageal transit for drug absorption, which may not be as effective compared to exogenous mucoadhesive excipients Increases the risk of local adverse effect Potential for systemic absorption via sublingual and buccal regions Limited practical appeal and may require taste masking	(Collings et al., 2002; Jacobsen et al., 2004; Zhang et al., 2008; Standring, 2020)
**Innovative pharmaceutical approaches**	**Stent-based drug delivery systems**	Stent placement may be temporary or permanent (e.g. palliative measure to relieve dysphagia) Drug eluting stents may facilitate local delivery of drugs to the esophagus May provide drug release over a prolonged period (weeks to months) Can be modified to facilitate passive drug release or stimuli-induced drug release (the latter may enhance treatment outcomes)	Requires surgical intervention for stent placement in the esophagus, which increases the risk of complications Not suitable for disease states that involve large or patchy areas of the esophagus Further studies required to assess efficacy and safety	(Guo et al., 2008, 2010; Hindy et al., 2012; Huang et al., 2015; Liu et al., 2015; Shaikh et al., 2015; Wang et al., 2015; Zhang et al., 2017a, 2017b; Jin et al., 2018; Vermeulen & Siersema, 2018; Xia et al., 2019; Fouladian et al., 2021; Ha et al., 2021; Prasher et al., 2021; Zhang et al., 2021)
	**Film-based drug delivery systems**	Bioadhesive May provide protection to the mucosal surface under the film May facilitate local delivery of drugs to the esophagus May provide drug release over a prolonged period (weeks)	Film placement in the esophagus generally requires surgical intervention Films that can be self-administered (e.g. EsoCap system) are not suitable for patients with swallowing difficulties Owing to the inconsistent results in the limited studies to date, further studies are required to optimize and validate this dosage for esophageal drug delivery	(Kumar Srivastava et al., 2016; Poghosyan et al., 2016; Tang et al., 2018; Krause et al., 2020)
	**Nanoparticulate-based drug delivery systems (NDDS)**	Potential for passive targeting, active targeting and triggered release strategies for esophageal drug delivery Nanoencapsulation may increase the efficacy and/or reduce the toxicity of drugs NDDS can be used to deliver a wide range of compounds NDDS may be combined with other dosage forms (e.g. embedded into esophageal stent devices)	Its application for esophageal drug delivery is still in the early investigational stage Of the limited studies available, the majority involve intravenous administration Further studies required to assess efficacy, targeting specificity, pharmacokinetics, safety, and patient tolerability	(Hua et al., 2018; Jiang et al., 2018; Lee et al., 2018; Xiao et al., 2019; Deng et al., 2020; Jun et al., 2020; Gao et al., 2021; Liu et al., 2021; Xu et al., 2021)

### Conventional liquid formulations for esophageal drug delivery

Conventional liquid formulations are the predominant topical dosage form on the market and in clinical trials to treat esophageal diseases (Table 2). Many of these formulations contain various types of polymers, polysaccharides, or glycerin to confer mucoadhesive properties and/or enhance the viscosity of the formulation to prolong transit time through the esophagus. These formulations have been used for drug delivery across the esophageal mucosa or as a protectant for the esophagus against potential irritants or injury (e.g. gastric acid) (Batchelor et al., 2002; Batchelor, 2005; Woodland et al., 2013). The advantages of liquid preparations are ease of administration and the ability to cover a large surface area when swallowed (depending on the dosage volume). To date, topical liquid formulations available on the market for the specific treatment of esophageal diseases are limited to sucralfate, liquid antacids, and combined formulation of antacid and sodium alginate – these are used to relieve the symptoms of reflux oesophagitis by neutralizing the luminal pH and/or protecting the esophageal mucosa from irritants in the gastric content. There are currently no topical liquid formulations on the market for drug delivery across the esophageal mucosa. Common compounds used to provide mucoadhesion and viscosity to liquid formulations for esophageal targeting are discussed below.

**Table 2.** Oral mucoadhesive formulations in clinical trials for topical esophageal drug delivery (Ref: clinicaltrials.gov).

Product	Study Size	Study Duration	Indication	Status	Year
Budesonide oral gel	36	9 weeks	Eosinophilic oesophagitis	Not yet recruiting	2023
Budesonide oral suspension	60	52 weeks	Esophageal strictures	Not yet recruiting	2023
Oral solution composed of hyaluronic acid, chondroitin sulfate, poloxamer 407 (Ziverel^®^)	200	52 weeks	Post operating esophageal relieve	Recruiting	2023
Alginate solution (Gaviscon Advance^®^)	24	78 weeks	Pre-operation	Completed	2022
Budesonide oral suspension	318	12 weeks	Eosinophilic Oesophagitis	Completed	2022
Budesonide oral suspension	133	313 weeks	Eosinophilic oesophagitis	Recruiting	2022
Hydrocortisone sodium succinate in aluminum phosphate gel	54	8 weeks	Esophageal strictures	Completed	2022
Sodium alginate solution	94	55 months	Protection	Completed	2021
Mometasone Furoate (hard gelatin capsule)	36	12 weeks	Eosinophilic Oesophagitis	Recruiting	2021
Sodium Alginate (Gaviscon Advance^®^)	30	Each subject had 3 visits, at least 24 hours apart	Reflux oesophagitis	Completed	2020
Hydrocortisone sodium succinate in aluminum phosphate gel	36	12 weeks	Esophageal strictures	Completed	2019
Hydrocortisone sodium succinate mixed with Aluminum Phosphate gel	66	13 weeks	Prevention of post-operative esophageal stricture	Completed	2019
Oral Budesonide Suspension	219	36 weeks	Eosinophilic oesophagitis	Completed	2019
Oral viscous budesonide and fluticasone MDI	129	9 weeks	Eosinophilic oesophagitis	Completed	2019
Oral viscous budesonide	48	17 months	Eosinophilic Oesophagitis	Completed	2018
Oral viscous budesonide	3	16 weeks	Eosinophilic oesophagitis	Terminated (recruitment was below expectation)	2018
Budesonide oral suspension	82	24 weeks	Eosinophilic Oesophagitis	Completed	2018
Oral viscous budesonide versus fluticasone MDI dipropionate	68	12 weeks	Eosinophilic Oesophagitis	Completed	2018
Oral cromolyn sodium viscous solution	16	2 months	Eosinophilic Oesophagitis	Completed	2018
Oral viscous budesonide	36	36 weeks	Eosinophilic Oesophagitis	Completed	2017
Sodium alginate oral suspension (Gaviscon^®^ sachet)	16	NA	GERD	Completed	2017
Suspension composed of oxetacaine, aluminum and magnesium hydroxide (Tepilta^®^)	40	11 weeks	Post operation esophageal relieve	Terminated (administrative reasons)	2017
Sodium alginate solution	Not reported	12 months	Oesophagitis	Completed	2017
Oral cromolyn sodium viscous solution	16	9 weeks	Eosinophilic oesophagitis	Completed	2017
Sucralfate slurry	3	4 weeks	Eosinophilic oesophagitis	Completed	2016
Hyaluronic acid, chondroitin sulfate and poloxamer 407 (Esoxx^®^)	154	4 weeks	GERD	Completed	2016
Alginate-based formulation (Gaviscon Liquid^®^) versus Magnesium-aluminum liquid antacid (Maalox^®^)	100	2 weeks	GERD	Completed	2016
Budesonide oral suspension	93	12 weeks	Eosinophilic Oesophagitis	Completed	2016
D-xylose suspension	14	52 weeks	Eosinophilic oesophagitis	Terminated (negative results)	2016
Sodium alginate oral suspension (Gaviscon^®^ sachet)	644	1 weeks	GERD	Completed	2016
Sodium alginate oral suspension	80	9 weeks	GERD	Completed	2015
Budesonide mixed with Splenda versus budesonide mixed with Neocate Nutra	60	6 months	Eosinophilic Oesophagitis	Completed	2014
Budesonide effervescent tablet and suspension	76	2 weeks	Eosinophilic oesophagitis	Completed	2014
Budesonide inhalation solution (respule) mixed with xanthan gum gel slurry	24	NA	Eosinophilic Oesophagitis	Completed	2014
Oral budesonide suspension	71	12 weeks	Eosinophilic Oesophagitis	Completed	2014
Oral Budesonide Suspension (MB-9)	93	24 weeks	Eosinophilic oesophagitis	Completed	2014
Oral viscous budesonide	6	4 months	Esophageal bullosa (Epidermolysis Bullosa)	Completed	2014
Oral viscous budesonide and fluticasone MDI	64	13 weeks	Eosinophilic oesophagitis	Completed	2013
Sodium alginate double action tablets	45	NA	GERD	Completed	2013
Alginate-simethicone xanthan gum solution	75	9 weeks	GERD	Unknown	2013
Sodium alginate oral suspension	195	4 weeks	GERD	Completed	2012
Budesonide oral suspension and budesonide MDI	25	8 weeks	Eosinophilic oesophagitis	Completed	2011
Oral viscous budesonide versus lansoprazole	13	3 months	Eosinophilic Oesophagitis	Completed	2010
Oral viscous budesonide suspension (MB-7)	82	12 weeks	Eosinophilic oesophagitis	Completed	2010
Oral viscous budesonide	32	13 weeks	Eosinophilic oesophagitis	Completed	2009
Hexylaminoevulinate loaded chitosan gel	4	NA	Barrett’s esophagus	Completed	2007
Fluconazole oral suspension	42	5 weeks	Esophageal candidiasis	Completed	1995

#### Sucralfate

Sucralfate is an aluminum hydroxide complex of sucrose octasulfate. It is a viscous liquid preparation with mucoadhesive properties and is indicated for the management of GERD, peptic ulcers, and duodenal ulcers (Orlando et al., 1987; Savarino et al., 2017a). Marketed formulations containing sucralfate act by adhering to the esophageal mucosal surface to provide a physical barrier against irritants from the gastric content, especially at sites of ulceration, which allows the affected tissues to heal (Orlando et al., 1987; Savarino et al., 2017a). The affinity of sucralfate for defective mucosa is based on the compound’s viscous nature as well as its ability to form polyvalent bridges between the cationic (positive charged) proteins present in high concentrations in the mucosal lesions and the anionic (negative charged) sucralfate polyanions (Nagashima, 1981). Sucralfate also buffers acid, inhibits the action of pepsin, and adsorbs bile salts (Nagashima, 1981).

Sucralfate liquid formulation is generally well tolerated and has relatively low absorption (0.5–2.2%) throughout the GI tract (Brogden et al., 1984). Two clinical trials compared the efficacy of sucralfate to H_2_ receptor antagonist, cimetidine, for patients with active reflux oesophagitis and concluded that sucralfate and cimetidine have beneficial treatment outcomes, which were comparable based on endoscopic evaluation (Tytgat, 1987; Jørgensen & Elsborg, 1991). In terms of effect on luminal pH, Orlando et al. (1987) evaluated the potential of esophageal protection in rabbit esophagus exposed to acid and reported that the pH of the luminal contents increased upon treatment with sucralfate and penetration of acid across the esophageal lining was markedly reduced. Furthermore, Slomiany et al. (1986) reported that sucralfate significantly increased the viscosity of porcine gastric mucus and reduced acid permeability by impeding hydrogen ions from penetrating the mucus. The findings from this study may apply to the esophageal mucosa and explain the protective effect of sucralfate on the esophageal lining.

Overall, sucralfate appears beneficial as a mucosal protectant; however, its use as a topical mucoadhesive base to deliver drugs would need further investigation. Based on its chemical structure, it may bind to compounds and prevent their absorption across the mucosa. In fact, the bioavailability of many drugs was reported to be reduced when co-administered with sucralfate (Sulochana et al., 2016). An advantage of this mucoadhesive compound is its ability to bind specifically to sites of mucosal injury, which would be beneficial to increase the delivery of therapeutic or diagnostic compounds to diseased/defective tissue and minimize accumulation in healthy mucosal tissue.

#### Alginate

Alginate is a natural, unbranched anionic polysaccharide that is found in algae (Lee & Mooney, 2012). The polymer is able to form a hydrogel via various methods such as ionic cross-linking with divalent cations (e.g. calcium ions) or covalent cross-linking with poly(ethylene glycol)-diamines (Eiselt et al., 1999). Those that have been modified with methacrylate and cross-linked with both eosin and triethanol amines also formed soft and viscoelastic hydrogels when exposed to laser (Smeds & Grinstaff, 2001). Alginates have gained interest for use as an esophageal protectant as well as in esophageal drug delivery due to its mucoadhesive property (Richardson et al., 2004; Batchelor, 2005; Woodland et al., 2015), which is predominantly determined by the length of the polymeric chain and the presence of ionizable groups (rather than on viscosity alone) (Woodland et al., 2015). For example, Batchelor et al. (Batchelor et al., 2002) investigated the use of alginate solution at various concentrations (2, 3 and 5% w/v) and across different molecular weights (MW 416, 387, 240, 220, 75 and 40) in *ex vivo* studies using the dynamic flow model on porcine esophageal tissue (length of 6 cm and width of 1.2 cm). The formulations prepared from higher molecular weight alginates generally showed an increase in retention time compared to lower molecular weight alginates. For instance, the 2% w/v alginate concentration (MW 416 and 387) showed ∼20–30% retention on the esophageal tissue after washing with artificial saliva at a rate of 1 mL/min for 30 minutes in comparison to the lower molecular weight alginates (MW 240 and 220, ∼15–35% remained; MW 75 and 40, ∼0–15% remained).

Similarly, Woodland et al. (2015) evaluated a marketed antacid liquid formulation containing sodium alginate (Gaviscon Advance®) for the degree and duration of its protectant effect on a 3D cell culture model resembling human esophageal mucosa. Gaviscon Advance^®^ consists of mainly sodium alginate and potassium hydrogen carbonate, followed by calcium carbonate, carbomer, and excipients including methyl and propyl parahydroxybenzoates (E218 and E216), sodium saccharin, sodium hydroxide, peppermint flavoring agent, and water (Reckitt Benckiser Healthcare (UK) Limited., 2002). *In vitro* studies using human esophageal epithelial cells demonstrated that barrier integrity (measured using transepithelial electrical resistance, TEER) was maintained in the cells coated with the alginate formulation prior to exposure to an acidic solution for 30 minutes (Krebs-Henseleit buffer at pH 3 + 0.5 mM taurodeoxycholic acid); however, the TEER significantly decreased by 58% and 62% for the cells coated with the viscous control formulation (glucose syrup and xanthan gum) and no treatment, respectively (Woodland et al., 2015). Similar results were seen in Ussing chamber assays on human esophageal biopsies, with pretreatment of tissues with alginate formulation for 5 minutes prior to exposure to an acidic solution for 30 minutes (Krebs-Henseleit buffer at pH 2 + 1 mg/mL porcine pepsin + 1 mM taurodeoxycholic acid) resulting in a minor reduction in TEER of 8.3% compared to the viscous control formulation (25% reduction) (Woodland et al., 2015). These combination antacid and sodium alginate formulations work not only by neutralizing acids in the esophageal lumen and stomach, but the sodium alginate also reacts with other components in the antacid (e.g. calcium, aluminum or magnesium) to form a floating gel raft that displaces acid pockets developed in the proximal stomach postprandial, thereby preventing reflux of the acid into the esophagus (Kwiatek et al., 2011; Woodland et al., 2015). In terms of safety, several studies have shown alginate to be nontoxic and biocompatible (Otterlei et al., 1991; Zimmemann et al. 1992; Lee & Lee, 2009; Lee & Mooney, 2012).

In addition to mucosal protection, alginate holds great potential for gastrointestinal drug delivery due to its mucoadhesive properties. The degree of mucoadhesion can potentially be controlled by altering the molecular weight and/or polymer length of the alginate as well as the use of various techniques to form the hydrogels (e.g. cross-linking agents, chemical methods, laser). Further studies are required to systematically evaluate the use of alginates for topical drug delivery in the esophagus, including variations in the alginate composition. This would determine the reproducibility in esophageal mucosal coverage, mucosal contact time, and mucosal drug permeability – factors important for clinical translation.

#### Poloxamer 407

Poloxamer 407 is a nonionic, hydrophilic triblock copolymer surfactant that exhibits thermoreversible properties (Dumortier et al., 2006; Fakhari et al., 2017). It consists of a central hydrophobic block of polypropylene glycol that is flanked on each side by polyethylene glycol blocks. This compound has been used as an ingredient in various pharmaceutical products and has been classified as being both biocompatible and inactive (Fakhari et al., 2017). Poloxamer 407 is a liquid at low temperatures but converts to a semi-solid gel state at room temperature or above. The gelation is due to aggregation of the copolymer molecules with formation of micelles that are arranged in an orderly manner (Dumortier et al., 2006). At concentrations between 15 to 30%, the solid-gel transition temperature is close to normal body temperature (Ricci et al., 2002; Giuliano et al., 2018), which potentially allows for sustained release of embedded bioactive compounds (Ricci et al., 2005; Giuliano et al., 2018). For example, Esoxx^®^ is a marketed formulation composed of hyaluronic acid, chondroitin sulfate and poloxamer 407 that is indicated for use in conjunction with other GERD treatment by forming a protective physical barrier (Di Simone et al., 2012; Savarino et al., 2017b). Hyaluronic acid and chondroitin sulfate have been suggested to stimulate angiogenesis and enhance wound healing (Di Simone et al., 2012; Savarino et al., 2017b).

Although poloxamer 407 has shown promising results for mucosal drug delivery, there are limited studies focusing on its application for esophageal drug delivery. For example, Antonino et al. (2019) demonstrated that 30% of the poloxamer 407 (16% w/w) formulation loaded with budesonide remained on the esophageal mucosal surface after four rinses with a buffer solution in an *ex vivo* study. Correspondingly, fluorescence tomography data showed mucoadhesion of the same formulation to the esophageal lining for at least 4 hours following oral administration in healthy male Swiss mice. This study demonstrated prolonged esophageal mucosal retention time with poloxamer 407, which could be beneficial for topical drug delivery in the esophagus. Further studies are required to assess the concentration dependent physicochemical properties of this polymer to ensure optimal and reproducible mucosal retention and drug absorption. Efficacy and safety studies in preclinical *in vivo* studies would also determine its potential for clinical translation. In addition, poloxamer 407 may be used in combination with other mucoadhesive compounds, but this has not yet been explored in detail for esophageal drug delivery.

#### Chitosan

Chitosan is a water soluble, polycationic polymer that exhibits antimicrobial activity and is biocompatible and biodegradable (Sinha et al., 2004; Martău et al., 2019). It is synthesized from chitin, which is usually sourced from crustaceans such as the shells of crabs, shrimps, and mollusks (Martău et al., 2019). Chitosan has shown promising mucoadhesive properties due to hydrogen bond, electrostatic, and ionic interactions between the charged amino groups in the polymer and the mucus (Fiebrig et al., 1995; Gåserød et al., 1998; Deacon et al., 2000). Adhesion of chitosan to mucus is dependent on the surrounding pH, the presence and amount of sialic acid residues, and the density of cross-linkage (He et al., 1998; Sandri et al., 2012). Chitosan tends to interact with sialic acid, which is a negatively charged sugar unit with a 9-carbon backbone found in the terminal of mucin (Sokolovskaya et al., 2022). Mucoadhesion of chitosan is improved when the polymer has a high charge density and the surrounding pH is <6 (He et al., 1998).

Chitosan has been investigated for potential use in esophageal drug delivery. For example, Collaud et al. (2007) compared chitosan with other oral formulations – i.e. poloxamer 407 (16, 17, 18 and 20% w/v), cross-linked polyacrylic acid (17% w/v), hydroxypropyl methylcellulose (HPMC, 1.8% w/v), and sodium carboxymethyl cellulose (NaCMC, 1% w/v) for delivery of hexaminolevulinate to the esophagus for diagnosis of Barrett’s esophagus. The chitosan formulations were prepared from chitosan with 1020 kDa (1.0% w/v) and 749 kDa (1.5 and 1.7% w/v). Chitosan 1020 kDa showed the highest mucosal retention (∼3.3 rinses to remove 50% of the formulation on rat esophagus) in *ex vivo* studies followed by chitosan 749 kDa (∼2.8 rinses), NaCMC (∼1.9 rinses), HPMC (∼1.3 rinses), cross-linked polyacrylic acid (∼0.5 rinses), and then poloxamer 407 (∼0.5 rinses). In clinical studies in healthy participants, the formulations (1.5% and 1.7% chitosan 749 kDa; 16%, 17%, 18% and 20% poloxamer 407; and 1.0% NaCMC gel) were tagged with E131 blue dye to allow assessment of mucosal retention and esophageal coverage. Ten minutes following oral administration, endoscopy showed that both chitosan (749 kDa) and NaCMC formulations gave complete esophageal surface coverage, whereas poloxamer 407 only gave partial coverage. The degree of retention was highest for 1.7% (w/v) chitosan (749 kDa) formulation. Additionally, no adverse effects were observed or reported for any of the formulations in this study.

Studies have demonstrated that chitosan, chitosan salt, and chitosan derivatives are able to enhance mucosal penetration in a dose dependent manner by loosening epithelial tight junctions (Opanasopit et al., 2007 Sadeghi et al., 2008; Canali et al., 2012). Although the effect of chitosan on esophageal mucosal permeability has not been investigated, it has been shown to increase permeability of drugs, proteins and peptides in *in vitro* and *ex vivo* studies on human colorectal adenocarcinoma cells (Kowapradit et al., 2010; Sonaje et al., 2012; Benediktsdóttir et al., 2014), non-small-cell lung adenocarcinoma cells (Casettari et al., 2010; Vllasaliu et al., 2010), porcine oral mucosa (Senel et al., 2000), human sigmoid colon tissue (Canali et al., 2012), rat colon (Canali et al., 2012), and porcine urinary bladder (Kos et al., 2006). The increase in mucosal permeability is thought to be due to chitosan-induced translocation of tight junction proteins (Ranaldi et al., 2002; Smith et al., 2004; Sonaje et al., 2012) and interference of the lipid organization in the mucosal epithelium (Senel et al., 2000). However, mucosal permeability is dependent on the molecular weight of chitosan, type of chitosan derivatives, pH of the surrounding environment, and charge density of chitosan (Kotzé et al., 1998; Opanasopit et al., 2007; Casettari et al., 2010).

Chitosan and its derivatives represent a promising group of mucoadhesive compounds for esophageal drug delivery, owing to its capability of covering the entire surface of the esophagus following administration in human participants. It would particularly suit esophageal conditions that require nonspecific coverage to both diseased and healthy tissue in the esophagus. Similar to other mucoadhesive compounds, further studies are required to determine the concentration-dependent mucosal retention and drug absorption of chitosan-based formulations for esophageal drug delivery. Efficacy data in preclinical studies would be warranted to support clinical translation. Chitosan has the advantages of known safety profile, being readily available, and extensive studies supporting its use in drug delivery – including for gastrointestinal drug delivery.

#### Carbomer

Carbomers are synthetic, high molecular weight, anionic polyacrylic acid cross-linked polymers (Brady et al., 2017). These polymers are acidic in nature and require neutralization with inorganic bases to achieve gelation (Kulkarni & Shaw, 2016). Gelation occurs as the polymeric chains within the carbomers become ionized and repel from each other, thereby causing the chains to uncoil (Mastropietro et al., 2017). Carbomers are used in various formulations as a thickener, stabilizer, and gelling agent. The first commercially available carbomers were developed by Lubrizol with the trademark Carbopol^®^. Carbopol® of different grades have been developed, with each differing by their physical properties as well as cross-linking method. Combination of carbomers have been used to achieve different formulation properties in solid, semi-solid, and liquid dosage forms.

There are limited studies available that have investigated the mucoadhesive property of carbomer formulations on the esophageal mucosa. For example, Bonacucina et al. (2004) prepared gel formulations containing carbomers (i.e. Carbopol^®^ 971 or 974) with water, polyethylene glycol 400 (PEG 400), or glycerin. Mucoadhesive strength was assessed on bovine esophagus by analyzing the maximum force required to separate the gel from the mucosa. Results showed that Carbopol^®^ 974 formulations with water (∼6.21 newton unit of force (N)) are more mucoadhesive compared to those prepared with PEG 400 (∼5.72 N) or glycerin (∼2.77 N). Similarly, Carbopol^®^ 971 formulated with water exhibited better mucoadhesive property (∼4.60 N) compared to PEG 400 (∼1.36 N) or glycerin (∼2.87 N).

In addition, Riley et al. (2001) investigated the esophageal and gastric mucosal retention times of different ^14^C labeled 3% polyacrylic acid formulations following oral administration in rats. The formulations used in this study were prepared from polyacrylic acid with either low (MW 140,000), high (MW 2,960,000), or ultra-high (MW 10^6^–10^9^) molecular weights to compare their effect on the mucoadhesive property of the formulations. Regardless of the molecular weight, all formulations were not retained in the esophagus at 15 minutes post administration. Following this *in vivo* study, Smart et al. (2003) conducted an *ex vivo* study using the same formulation parameters on porcine esophageal mucosa and gastric mucosa. Results showed that formulations comprised of high molecular weight polyacrylic acid (MW 2,960,000) were able to bind to the esophageal and gastric mucosa for an extended period – i.e. ∼38% remained on esophageal and fundus tissue and ∼63% remained on pyloric tissue after continuous flushing with 1% hydrochloric acid solution at a flow rate of 1 mL/min for 20 minutes (Smart et al., 2003). The low molecular weight and ultra-high molecular weight formulations were inferior to the high molecular weight formulation, with ∼22% and ∼31% remaining on esophageal tissue, ∼3% and ∼22% remaining on fundus tissue, and ∼13% and ∼24% remaining on pyloric tissue, respectively. The difference in *in vivo* and *ex vivo* findings were suggested to be due to the presence of keratinization of esophageal epithelium in rats, turn-over of mucus in the tissue, or the amount of polymers that had adhered to the rat esophagus was too low to be detected (Smart et al., 2003). Interestingly, formulation distribution in this *ex vivo* study was reported to be uneven throughout the length of the esophagus (length of 15 cm and width of 4 cm), regardless of the molecular weight of the polyacrylic acid.

The use of carbomers for topical drug delivery to the esophagus has potential. Further studies are needed to assess reproducibility in esophageal coverage, mucosal retention, and drug absorption as well as comprehensive data evaluating efficacy in preclinical studies. In addition, there are a lack of studies comparing carbomers with other mucoadhesive polymers for esophageal drug delivery. For example, some studies have reported carbomers to be superior to chitosan in terms of mucoadhesion (Kockisch et al., 2003; Collaud et al., 2007). It is likely that mucoadhesion of carbomer formulations to mucosal tissue is influenced by multiple factors such as the interaction of the formulation with mucin as well as the interactions between the polyacrylic acid and water molecules.

#### Xanthan gum

Xanthan gum is a high molecular weight, natural, anionic polymer composed of chains of monosaccharides and oligosaccharides (García-Ochoa et al., 2000). The polymer is an extracellular secretion from *Xanthomonas campestris*, an aerobic gram-negative bacterium that is known to cause plant diseases (García-Ochoa et al., 2000). Xanthan gum is insoluble in most organic solvents, but dissolves in water to form a viscous solution that has high pseudoplasticity (Hublik, 2012). The polymer has the advantage of being stable across a wide range of parameters, including temperature (0–100 °C) and pH (Hublik, 2012). Xanthan gum is generally considered safe for human consumption and is used as a thickening agent in liquid pharmaceutical formulations for patients with dysphagia (Hefner et al., 2016). However, the use of xanthan gum in infants, especially those born prematurely is associated with increased risk of developing necrotizing enterocolitis, possibly through accumulation of short-chain fatty acid produced from digestion of the polymer by the gut microbiome (Sun et al., 2022). Xanthan gum has been reported to have weak mucoadhesive properties due to its high molecular weight that limits its penetration though the mucus layer, and the electrostatic charge repulsion between the polymer and mucin (Sosnik et al., 2014). Some earlier studies have reported that the mucoadhesiveness of xanthan gum is comparable to Carbopol® 934 and is better than hydroxypropyl cellulose (Sosnik et al., 2014). The mucoadhesiveness of xanthan gum may be improved via chemical modification (Patel et al., 2020).

Several studies have investigated the potential use of xanthan gum for topical drug delivery to the esophagus. For example, Hefner et al. (2016) conducted a clinical study to compare the esophageal retention time of liquid budesonide formulations in healthy participants for the potential treatment of EoE. The formulations were prepared by mixing budesonide respule (micronized particles) suspension with either honey, xanthan gum powder, or sucralose powder and Tc-99m sulfur colloid for nuclear scintillation imaging purposes. The esophageal contact of each formulation was quantified by area under the curve (AUC). The formulation containing xanthan gum showed significantly higher contact to esophageal mucosa (AUC∼50,000 at 1 min, AUV∼ 70,000 at 2 min, and AUC ∼90,000 at 3 min) compared to honey (AUC∼50,000 at 1 min, AUC∼60,000 at 2 min, and AUC ∼70,000 at 3 min) and sucralose formulations (AUC ∼43,000 at 1 min, AUC ∼50,000 at 2 min, and AUC ∼60,000 at 3 min). Based this study, Bonnet et al. (2018) developed a viscous liquid budesonide formulation composed of budesonide, xanthan gum, glycerol, EDTA, sodium saccharin, sodium benzoate and raspberry flavoring agent that remained stable for at least 3 months when stored at low temperatures (2–8 °C). It should be noted, however, that pharmacokinetics and efficacy were not analyzed in either study but are warranted, especially in patients with EoE, in which results may differ due to the pathophysiological changes and presence of symptoms such as dysphagia.

Limited studies have investigated the combination of xanthan gum with other natural polymers for esophageal drug delivery. Xanthan gum has been reported to interact with galactomannans such as guar gum, glucomannan, locust bean gum, or carrageenan to form viscous liquids and gels with elasticity cohesiveness as well as thermal reversible properties (Hublik, 2012; BeMiller, 2019; Sworn, 2021). Casiraghi et al. (2020) evaluated the *ex vivo* mucosal retention time on porcine esophageal tissue of various formulations of viscous budesonide liquid formulation using xanthan gum and guar gum at different ratios with other excipients such as sodium saccharin, glycerin, ethylenediaminetetraacetic acid, sodium benzoate, and water. The results showed that the formulation containing both xanthan gum (2.4% w/v) and guar gum (2.4% w/v) remained on the mucosal surface for up to 29 minutes and exhibited higher viscosity (204.46 pascal second (Pas) at 100 s^−1^ shear rate) compared to formulations containing 1.8% w/v xanthan gum and 1.8% w/v guar gum (135.60 Pas), 4.8% w/v xanthan gum (144.83 Pas), and 3.6% w/v xanthan gum (91.42 Pas). Importantly, regardless of formulation, this study highlighted that <1% of the budesonide in the formulation was absorbed into the porcine esophageal tissue.

Xanthan gum formulations have demonstrated prolonged esophageal mucosal retention time, which is recognized as one of the key requirements for effective topical drug delivery to the esophagus and for improved treatment outcomes. However, further studies are required to evaluate the pharmacokinetics and efficacy of these formulations for clinical translation. Combination of xanthan gum with other polymers such as guar gum may provide additional benefits; however, these formulations are still in the investigational stage and require further validation for esophageal drug delivery. Additional studies to comprehensively assess the safety of xanthan gum is warranted following its association with increased risk of gut inflammation in infants.

#### Thiolated hydroxypropyl-β-cyclodextrin

Thiolated polymers have been shown to have mucoadhesive properties as they are able to form disulfide bonds with glycoproteins in mucus (Perrone et al., 2017; Laquintana et al., 2019). For example, Laquintana et al. (2019) synthesized thiolated hydroxypropyl-β-cyclodextrin and evaluated its efficacy in delivering budesonide to the esophagus as a potential treatment of EoE. Budesonide was complexed with thiolated polymer through freeze drying and the liquid formulation (1 mg/mL) was prepared with addition of phosphate buffered saline (PBS). *Ex vivo* studies were performed using the dynamic flow model on porcine esophageal tissues that were continuously washed with PBS at a flow rate of 1 mL/min. Results showed that the liquid drug formulation had enhanced mucosal retention, with approximately 10% remaining at the 60-minute time point compared to the free drug control and drug free thiolated hydroxypropyl-β-cyclodextrin that were washed off by the 30 to 40 minutes time point. Cytotoxicity studies of thiolated hydroxypropyl-β-cyclodextrin on human colorectal carcinoma cell lines showed viability of ∼80% and 75–80% at 3- and 24-hours post-treatment, respectively. Interestingly, the cell viability was ∼90% when treated with hydroxypropyl-β-cyclodextrin, indicating that the thiolated compound exhibited higher cytotoxicity. At this stage, it is unclear whether the mucoadhesive properties of this complex translates to enhanced drug permeability into the esophageal mucosa or *in vivo* efficacy and safety. These studies are warranted to assess its potential for clinical application.

### Orodispersible tablets for esophageal drug delivery

Orodispersible tablets are also known as oral disintegrating or fast disintegrating tablets. It is an oral dosage form that incorporates effervescent characteristics that facilitates disintegration of the tablets within the oral cavity when in contact with saliva and does not require water for administration (Bandari et al., 2008). The disintegration time ranges from 15 seconds to 20 minutes, depending on the composition of the tablets (Ghosh et al., 2011). With stimulus from the effervescent effect, saliva secretion is increased and is mixed with the drug molecules before being swallowed. The innate mucoadhesive properties of saliva slows esophageal transit, allowing drugs to be absorbed into the mucosa. The effervescent effect of orodispersible tablets is created by a reaction between acids (e.g. citric acid, tartaric acid, malic acid, fumaric acid or succinic acid) with sodium hydrogen carbonate or sodium bicarbonate upon contact with water or saliva within the oral cavity (Al-Khattawi & Mohammed, 2013). Liberation of carbon dioxide from the reaction leads to disintegration of the tablet and release of the active pharmaceutical ingredients (Al-Khattawi & Mohammed, 2013).

Presently, budesonide orodispersible tablet (BOT) is the only commercially available formulation for esophageal drug delivery and is indicated for the treatment of EoE (Miehlke et al., 2020). Table 3 presents the summary of clinical studies that have investigated the efficacy of BOT for esophageal drug delivery. This formulation has shown promising results in recent clinical trials (Miehlke et al., 2016; Lucendo et al., 2019; Straumann et al., 2020; Miehlke et al., 2022). For example, Miehlke et al. (2016) compared the efficacy and safety of BOT and oral viscous budesonide suspension for 2 weeks in a double-blind, double-dummy, randomized controlled trial (*n* = 19 participants with active EoE per group) and reported no significant difference between the two formulations. Histological remission was achieved in ∼94.7 to 100% of the participants. In terms of adverse effects, no changes in baseline cortisol levels were detected in patients that received either BOT or oral viscous budesonide and development of oropharyngeal candidiasis was reported in ∼10.5% of patients in both treatment groups. A higher preference for BOT (80%) compared to the oral viscous budesonide formulation (13%) was reported in the study. In a subsequent clinical trial in a large cohort of patients with clinico-histological active EoE (*n* = 181), BOT (1 mg twice daily for 6 weeks) achieved clinico-histological remission in 69.6% of patients. Interestingly, deep endoscopic remission was achieved in only 53.6% of patients (Miehlke et al., 2022).

**Table 3.** Orodispersible tablet formulations in clinical trials for topical esophageal drug delivery (Ref: clinicaltrials.gov).

Product	Study Size	Study Duration	Indication	Status	Year
Fluticasone propionate orally disintegrating tablets	106	52 weeks	Eosinophilic Oesophagitis	Completed	2022
Fluticasone propionate orally disintegrating tablets	24	8 weeks	Eosinophilic Oesophagitis	Completed	2020
Budesonide orodispersible tablet	204	48 weeks	Eosinophilic Oesophagitis	Completed	2020
Budesonide orodispersible tablet	204	48 weeks	Eosinophilic oesophagitis	Completed	2020
Budesonide orodispersible tablet	87	12 weeks	Eosinophilic Oesophagitis	Completed	2019
Budesonide orodispersible tablet	88	6 weeks	Eosinophilic Oesophagitis	Completed	2019
Budesonide orodispersible tablet	88	6 weeks	Eosinophilic oesophagitis	Completed	2016
Budesonide effervescent tablet vs. suspension	76	2 weeks	Eosinophilic oesophagitis	Completed	2014

The pharmacokinetics of BOT was investigated by Dilger et al. (2013) in a phase I study involving 12 EoE and 12 healthy subjects. This study showed that the systemic exposure of budesonide (reflected through maximum plasma concentration (C_max_)) after administration of 1 mg, 2 mg and 4 mg BOT and 3 mg budesonide capsule were 0.44 ± 0.31 ng/mL, 0.90 ± 0.68 ng/mL, 1.89 ± 1.25 ng/mL and 0.72 ± 0.55 ng/mL, respectively, suggesting comparable bioavailability for both formulations. Despite the prolonged budesonide elimination half-life in EoE patients, no change in baseline cortisol level was detected in either EoE patients or healthy subjects that received 4 mg BOT or 3 mg budesonide capsule after once daily dosing for 7 days. These data suggest minimal systemic bioavailability of budesonide; however, this should be considered relative to budesonide having high first-pass metabolism.

Orodispersible tablets are a relatively new conventional formulation approach for esophageal drug delivery. Due to their rapid disintegration and dissolution, they are more commonly used for drug delivery in the oral cavity (e.g. sublingual or buccal) or for drugs requiring rapid systemic absorption in the small intestine (Bandari et al., 2008). Orodispersible tablets have shown promising results for topical esophageal drug delivery in EoE and could potentially be used for the treatment of other esophageal diseases. However, further studies are required to assess the esophageal mucosal retention time, degree of esophageal mucosal surface coverage, and depth of mucosal drug penetration of this type of conventional formulation, as these factors are important for the local treatment of esophageal diseases. In addition, orodispersible tablets rely on saliva as the mucoadhesive component to slow transit time through the esophagus, which may not be as effective compared to formulations containing exogenous mucoadhesive excipients. Evaluation of orodispersible tablets containing drugs that do not undergo high first-pass metabolism is also warranted to provide a better indication of the overall systemic exposure of this formulation.

### Lozenges, pastilles, and troches for esophageal drug delivery

Lozenges, pastilles, and troches are dosage forms that do not require water for administration and are retained within the oral cavity for gradual disintegration and dissolution in saliva (∼30 minutes) (Zhang et al., 2002). These formulations have been investigated for potential use in esophageal drug delivery, as the release of drugs into the saliva in the oral cavity can be swallowed to coat the esophagus. Medicated lozenges available commercially include those containing antiseptic, anesthetic, antimicrobial, and antifungal drugs that are indicated for the treatment or symptomatic relief of infections in the pharynx (Williams et al., 2007). The value of these commercially available formulations in esophageal diseases is not well established.

There are very few studies available that have investigated the use of lozenges, pastilles, or troches for esophageal drug delivery (Table 4). For example, Valenzano et al. (2014) evaluated the effectiveness of zinc lozenges for the prevention of esophageal carcinoma in a clinical study on patients with Barrett’s esophagus. Zinc deficiency has been reported to increase the risk of esophageal carcinoma (Grotenhuis et al., 2011; Li et al., 2014; Ma et al., 2018). Following treatment with the zinc lozenges for two weeks, atomic absorption analysis showed ∼30% increase in zinc levels in esophageal biopsy samples (∼0.23 µg zinc per mg of protein) compared to the placebo group (∼0.17 µg zinc per mg of protein) (Valenzano et al., 2014). However, it was suggested in other studies looking at zinc transporter 1 (Znt1) levels in biopsy samples and *in vitro* studies on Barrett’s epithelial cell cultures that the zinc released from the lozenges were not taken up by the epithelial cells and likely remained within the extracellular space (Liuzzi et al., 2001; Hara et al., 2017; Nishito & Kambe, 2019). In contrast, the potential prophylactic efficacy of zinc lozenges was reported in other studies, which showed significant decrease in pro-inflammatory and epithelial-mesenchymal transition (EMT) signaling at the gene and protein level within esophageal mucosal tissue after 2 weeks of treatment (Valenzano et al., 2021). The reason for the differing results will need to be investigated, including confirmation of cellular uptake of zinc in the epithelial cells.

**Table 4.** Lozenge, pastille, and troche formulations in clinical trials for topical esophageal drug delivery (Ref: clinicaltrials.gov).

Product	Study Size	Study Duration	Indication	Status	Year
Zinc gluconate lozenges	8	2 weeks	Barrett’s esophagus	Completed	2014
Flavored anesthetic lozenge	191	NA	Pre-operation	Completed	2010
Amphotericin B lozenge	40	3 weeks	Protection	Completed	2009

Lozenges, pastilles, and troches are well-established conventional dosage forms, but it is likely that their application for the treatment of esophageal diseases will be minimal owing to the limitations of these formulations. As disintegration and dissolution of these formulations are occurring in the oral cavity over an extended period, there is significant potential for dilution of the drug concentration that is able to reach the esophagus as well as for systemic absorption via the sublingual and buccal regions. Inconsistent drug release profiles may occur based on administration technique (i.e. sucking, chewing, or crushing the dosage form will release the drug more quickly) and the hydration status of the oral cavity (especially patients with chronic dry mouth due to concurrent medications or disease). In addition, not all types of drugs are suitable to be delivered via lozenge, pastille, or troche formulations due to their solubility, stability, pH, or pharmacodynamics. Some drugs could also cause staining in the oral cavity, be unpalatable due to bitterness, or cause local adverse effects (e.g. irritation to the mucosa).

### Chewing gums for esophageal drug delivery

Medicated chewing gums are dosage forms that incorporate drugs within a gum base. For esophageal drug delivery, the active compound is gradually released and mixed with saliva as the gum is chewed. Although the gum should not be swallowed, it is the swallowing of the saliva containing drug that allows coating of the esophageal mucosa (Zhang et al., 2008). There are currently no marketed formulations and one clinical trial of chewing gum-based therapies for the treatment of esophageal diseases. For completeness, a chewing gum formulation containing sildenafil was previously patented for the treatment of esophageal spasm (Standring, 2020). However, the formulation was not translated into a commercial product and the patent expired in April 2019. An antacid chewing gum (Surpass^®^) was marketed in 2001 as a treatment for heartburn and acid reflux, but the product was withdrawn two years later due to lack of popularity (Zhang et al., 2008). Surpass^®^ was composed of 300 mg calcium carbonate embedded in acacia gum base along with other inactive excipients (Company WWJ. Surpass® Antacid Gum., 2000). Collings et al. (2002) compared the efficacy of antacid chewing gums and chewable antacid tablets for the treatment of heartburn. The results showed greater increase in esophageal pH after administration of the antacid chewing gum (pH 6.5 to 7) compared to chewable antacids tablets (pH 6.0 to 6.5). In addition, the pH neutralizing effect of the chewing gum formulation (esophageal pH remained at 6.0 for 20 minutes after administration) was more prolonged compared to the chewable tablets (pH 5.0 for 20 minutes after administration). Currently, there is only one ongoing clinical study evaluating the use of calcite chewing gum for the treatment of GERD (clinical trial identifier: NCT05129670).

As a dosage form for esophageal drug delivery, chewing gums appear to have limited clinical application as well as practical appeal. Although it does not require water for administration and the effect can be terminated by removing the gum, this drug delivery strategy would be difficult to attain consistent and reproducible drug concentrations at the target site. Drug release is influenced by the chewing technique and the drug itself may also remain adhered to the gum base rather than being released into the saliva (Jacobsen et al., 2004). In addition, similar to lozenges, pastilles, and troches, chewing gum formulations are in the oral cavity for a prolonged duration which increases the risk of local adverse effects as well as systemic absorption from the buccal and sublingual regions (Jacobsen et al., 2004). Taste masking is also required in chewing gum formulation to increase palatability and improve patient compliance.

## Innovative pharmaceutical approaches for topical drug delivery to the esophagus

**Figure 3.**
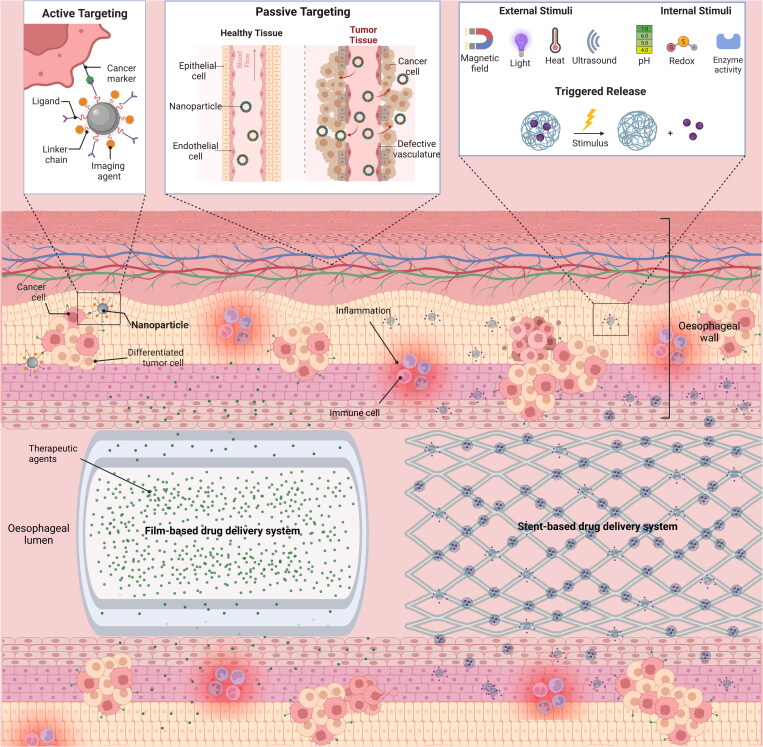
Schematic diagram of the innovative pharmaceutical approaches for esophageal drug delivery.

Innovative pharmaceutical approaches have been developed to overcome the physiological and pathophysiological obstacles to effective topical drug delivery in the esophagus. These approaches aim to improve mucosal retention time and drug permeability across the esophageal mucosa to a greater extent than can be achieved with conventional formulations. This section will discuss the main innovative pharmaceutical approaches, namely stent-based, film-based, and nanoparticle-based esophageal drug delivery systems (Figure 3) (Table 1). The development and effectiveness of the approaches will be discussed as well as the current status of the dosage form in the translational pipeline.

### Stent-based esophageal drug delivery systems

Esophageal stents are small, meshed tubes made of either metal (e.g. nitinol and stainless steel) or synthetic material (e.g. polyester, silicone, and polyurethane) that are inserted into the lumen of the esophagus (Figure 3). Stent placement may be temporary or permanent, and this procedure has been used primarily as a palliative measure to relieve dysphagia associated with various conditions (e.g. esophageal cancer, strictures, esophageal anastomoses leak, and esophageal perforations) (Hindy et al., 2012; Vermeulen & Siersema, 2018). Drug eluting stents have gained increasing attention in recent years for their ability to facilitate local delivery of drugs to the esophagus. The release rate of therapeutic agents can be controlled by altering the polymer binding of drugs to the stent. Many esophageal self-expanding stents have been investigated for the delivery of chemotherapeutic agents for the treatment of esophageal cancer (e.g. docetaxel (Huang et al., 2015; Shaikh et al., 2015; Fouladian et al., 2021), paclitaxel (Zhang et al., 2017b; Jin et al., 2018; Xia et al., 2019), 5-fluorouracil (Guo et al., 2010; Liu et al., 2015; Wang et al., 2015), radioactive seeds (e.g. Iodine^125^ (Guo et al., 2008,Zhang et al., 2021)), and cytotoxic polymers (e.g. polyethyleneimine (Zhang et al., 2017a)). Stents coated with biological materials (e.g. decellularized extracellular matrix (Ha et al., 2021)) and corticosteroids (e.g. fluticasone (Prasher et al., 2021)) for the treatment of oesophagitis have also been studied. Table 5 summarizes the stent-based esophageal drug delivery devices in clinical trials for topical esophageal drug delivery. It should be noted that complications associated with esophageal stent placement are common, including persistent chest pain (4.3–30%), recurrent dysphagia due to stent migration (11–24.5%), tissue ingrowth or overgrowth (2.2–14%), severe bleeding (3–8%), food obstruction (2.2–7.0%), GERD symptoms (2.6–7.0%), aspiration pneumonia (1.3–5.0%), and perforation (1.3–2.0%) (Hindy et al., 2012; Vermeulen & Siersema, 2018).

**Table 5.** Stent-based esophageal drug delivery devices in clinical trials for topical esophageal drug delivery (Ref: clinicaltrials.gov).

Product	Study Size	Study Duration	Indication	Status	Year
125I particle loaded esophageal stent	45	21 days	Esophageal cancer	Completed	2021
Infrared radiation responsive esophageal stent covered with doxorubicin loaded gold nanoturf	12	4 weeks	Esophageal cancer	Completed	2018
Iodine 125 seeds loaded esophageal stent	53	8 weeks	Esophageal cancer	Completed	2008

Presently, there are two main types of drug eluting stents that have been investigated – those that facilitate passive drug release or stimuli-induced drug release (e.g. heat generated from light or laser induction, or electromagnetic induction). An example of the former approach includes combination paclitaxel and 5-flurouracil eluting stent developed by Liu et al. (2015). The ethylene-vinyl acetate (EVA) coated nitinol stent showed an accumulated *in vitro* release of ∼10.8% for paclitaxel and ∼58.1% for 5-fluorouracil at day 13. The drug release rate reduced and became linear after day 13, with ∼20.6% paclitaxel and ∼92.0% 5-fluorouracil released at day 95 of the study. In the corresponding preclinical study in pigs, similar results were seen in which the esophageal tissue concentration of paclitaxel and 5-fluorouracil was highest on day 13, followed by a time dependent decrease in release rate. Drug accumulation of paclitaxel and 5-fluorouracil at day 13 was primarily in the esophagus (81.5 ± 9.48 μg/g and 58.3 ± 68.5 μg/g, respectively), with the drug concentrations in the plasma and other harvested organs (heart, liver, spleen, kidney, lung) detected at extremely low levels. For example, the average concentration of paclitaxel and 5-fluorouracil in the esophageal tissue (81.5 µg/g) was ∼2700-fold and 269-fold higher than that in the liver (0.03 µg/g and 0.217 µg/g), respectively. These results are likely due to the unidirectional and sustained drug release of this dosage form; however, high variability was identified with the 5-fluorouracil accumulation in the esophagus. As further support, Wang et al. (2015) evaluated the same formulation in a porcine model. No significant haematological changes or toxicity were detected up to 120 days post-stent implantation. Importantly, the study showed that the drug eluting stent was able to facilitate local and sustained release of paclitaxel and 5-fluorouracil in the esophagus for up to 95 days, with minimal drug accumulation in other organs.

Stimuli dependent drug eluting stents are usually composed of nitinol stents treated with photoactivated (e.g. gold) or magnetic materials (e.g. ferrum) and drug loaded polymer coatings (Jin et al., 2018; Lee et al., 2018). When induced with infrared or electromagnetic field (EMF) of a certain wavelength, heat is generated by the stent to cause structural or conformational changes to the drug loaded polymer coating, resulting in drug release. These stents exerts exothermic or photothermal effects which can enhance cancer therapy (Zou et al., 2016). Improved drug penetration across the esophageal mucosa has also been reported. For example, Jin et al. (2018) combined magnetocaloric nitinol stent (nitinol alloyed with ferrum) coated with EVA film containing 10% (w/w) paclitaxel and 30% (w/w) 1-hexadecanol (EVA-PTX-1H). The *in vitro* cumulative drug release from EVA-PTX-1H on day 15 with and without EMF induction (∼0.1 kW) was ∼16% and ∼11%, respectively. *In vivo* results in healthy rabbits showed an accumulative drug release with and without EMF induction of 49.85 ± 0.89% and 40.52 ± 2.02% on day 15, which is significantly higher compared to the *in vitro* data. EMF induction also improved the permeability of the drug across the esophageal mucosa (3.84 ± 0.77 μg per gram esophageal tissue with EMF and 1.46 ± 0.29 μg/g without EMF). Histological examination did not show any signs of inflammation, fibroblast proliferation, tissue granulation, or vascularization of the local tissue in contact with the coated stent, irrespective of EMF exposure. However, significant inflammation and granulation were observed in the tissue that was in contact with the uncoated surface of the stent. The same findings, except for slightly thickened basal cell layer, were observed in animals treated with the coated esophageal stents by Liu et al. (2015) and Wang et al. (2015) – thereby suggesting that coated stents cause significantly less tissue irritation. This could potentially reduce the risks of esophageal stent related complications. No signs of significant toxicity were identified following histological assessment of samples extracted from the heart, lungs, liver, spleen, and kidney from the animals in the study.

Overall, drug eluting stents may offer benefits as a therapeutic delivery system for esophageal pathologies based on their ability to localize drug accumulation to esophageal tissue over a prolonged duration (weeks to months). In addition, the medical device can be altered to support targeted radiotherapy or photodynamic therapy to enhance treatment outcomes. The efficacy, safety, risks, and complications of these stents for long term use require further evaluation and validation in *in vivo* models of esophageal disease. It would also be important to determine whether topical drug accumulation is restricted to the esophageal tissue that is in direct contact with the stent, as this would have limited use for disease states that involve large or patchy areas of the esophagus.

### Film-based dosage forms for esophageal drug delivery

Polymeric films have gained increasing interest for potential use in drug delivery to specific regions in the GI tract (Hua, 2020). These films are composed of either natural polymers (e.g. gelatin, carrageenan, chitosan, collagen, hyaluronic acid, sodium alginate, and guar gum) and/or synthetic polymers (e.g. carboxymethylcellulose (CMC), HPMC, polyvinyl alcohol (PVA), polyglycolic acid, polycaprolactone (PCL), polyvinylpyrrolidone (PVP), and poly(d,l-lactide-co-glycolate) (PLGA)) (Hua, 2020). The adhesive and porous nature of these polymeric films allows for good adhesion to mucosal surfaces, which can provide protection as well be loaded with therapeutic agents (e.g. drugs, collagen, various glycoproteins, and growth factors) for sustained drug release (Poghosyan et al., 2016; Tang et al., 2014; Kilicarslan et al., 2018; Tang et al., 2018; Pamlényi et al., 2021).

Recent developments in esophageal drug delivery have adopted film-based dosage forms for the treatment of esophageal diseases (Figure 3). For example, Krause et al. (2020) developed the EsoCap system which is an orally administered dosage form consisting of a sinker device, a trainer, and a rolled-up drug-loaded PVA film (22 cm long and 0.4 cm wide) stored within a slitted gelatin capsule. A food grade polyester thread that connects the PVA film and the EsoCap applicator remains intact while swallowing, allowing the film to unroll within the esophagus as the capsule transits through the organ. It is recommended to detach the thread from the applicator at least 2 minutes after administration to ensure sufficient time for the film to adhere to the esophageal mucosa (i.e. the patient may either swallow or pull the thread out of the throat, leaving the PVA film in the esophagus). Krause et al. (2020) conducted a proof-of-concept clinical study on 12 healthy participants and reported an average swallowability score of 20 out of 100 (*n* = 36) (0 is without discomfort while swallowing and 100 is very difficult to be swallowed) without nausea or vomiting. The main source of discomfort was from the thread connecting the film and applicator. Magnetic resonance imaging confirmed that the PVA film rolled out successfully after administration (∼100%) (*n* = 36), and an *in vitro* dissolution test showed that 80% of an embedded fluorescent dye was released from the PVA film after 25 minutes (with complete release within 1 hour). Rosenbaum et al. (2021) evaluated an improved version of the EsoCap system, which has an additional sinker in the capsule to enhance its swallowability. However, the average swallowability score for the new EsoCap system (22 out of 100) was not significantly different from the earlier version (Rosenbaum et al., 2021).

Polymeric films have also been investigated to augment wound healing in the esophagus following surgical interventions to prevent stricture formation. For example, Tang et al. (2018) developed a film like ‘sheet’ composed of CMC and hyaluronic acid. In preclinical studies in pigs, the sheets were applied topically via endoscopic forceps following submucosal dissection. Although the treatment group (CMC sheets) showed slightly better tolerability toward food 7 days post-surgery compared to the control group (no treatment), macroscopic examination revealed no significant difference in stricture formation between the study groups (i.e. all pigs had strictures by day 14). The CMC sheet was not seen on the wound surface at day 7, which suggests degradation of the polymer film.

Another example of film-based dosage form to improve esophageal wound healing is the fibroblast growth factor (FGF) loaded gelatin film developed by Fedakar-Senyucel et al. (2008). The formulation was sutured to esophageal anastomoses induced in Wistar albino rats and the treatment efficacy was evaluated for 7 days. Results showed significantly faster wound healing and increased wound break strength (mean bursting pressure of 62.3 ± 6.8 mmHg) in the treatment group compared to placebo (gelatin film without FGF; 29.0 ± 1.6 mmHg) and control group (no treatment; 22.5 ± 3.1 mmHg). Correspondingly, the mean submucosal collagen deposition score and mean tissue hydroxyproline concentration (major amino acid of collagen) in the treatment group were significantly higher (1.5 ± 0.2 and 6.0 ± 1.0 µg/mg) compared to placebo (0.7 ± 0.1 and 3.9 ± 0.4 µg/mg) and control (0.7 ± 0.2 and 2.4 ± 0.5 µg/mg) (Fedakar-Senyucel et al., 2008), indicating enhanced cell proliferation and improved wound healing (Kumar Srivastava et al., 2016).

Film-based drug delivery system are an innovative dosage form that has been investigated for potential use in the treatment of esophageal diseases. The studies to date show the limitation of this dosage form for self-administration, especially in patients with swallowing difficulty, with many likely limited for surgical use. Despite the inconsistent results in the limited studies to date, further studies are required to optimize and validate this dosage form for clinical translation. This includes validation of reproducible adhesion of the film in the esophagus over a specific duration as well as adequate drug release and absorption into the esophageal tissue. Incorporation of drugs and other bioactive compounds could potentially improve surgical treatment outcomes (e.g. improve wound healing or reduce risk of infections) as well as treat other more serious esophageal diseases; however, efficacy and safety studies are warranted in preclinical esophageal disease models to determine its place in therapy. Lastly, with regard to the EsoCap system, there are still gaps to bridge for this system to be ready for clinical use. In addition to the studies recommended above, further optimization may be required to improve the swallowability of the dosage form to enhance patient compliance. At this stage, this method of administration is likely unsuitable for children and patients with swallowing difficulties.

### Nanoparticulate-based dosage forms for esophageal drug delivery

Nanoparticulate-based drug delivery systems (NDDS) have been widely investigated in the past few decades for the diagnosis and treatment of diseases (Hua et al., 2018). The use of NDDS for esophageal targeting is still in the early investigational stage. Of the limited studies available, the majority involve intravenous administration, with only a few studies focusing on topical application. As highlighted earlier in this review, the esophagus poses a significant challenge for drug delivery, especially topical drug delivery, due to the biological barriers that makes this organ relatively impermeable to compounds. Nanoencapsulation can provide a strategy to increase the efficacy and/or reduce the toxicity of drugs (Hua et al., 2018). It can also be used to deliver compounds which have physicochemical properties that strongly limit their aqueous solubility and/or degrade easily in biological environments (Hua et al., 2018).

The following section discusses the different NDDS approaches that have been evaluated for esophageal drug delivery. Both systemic and topical routes of administration have been included to provide a more comprehensive understanding of the potential place of nanoparticle therapy for the management of esophageal diseases (Figure 3). In general, the main NDDS approaches for drug delivery to specific tissues or organs are passive targeting, active targeting, and triggered release (Hua et al., 2015, 2018; Sercombe et al., 2015). Passive targeting relies on physicochemical properties such as size, surface charge, and/or surface coating (e.g. with polyethylene glycol (PEG)) with components that are not ligands for specific tissue or organ binding to enhance drug accumulation in target regions (i.e. specific organs, tissues, cells) (Hua et al., 2015, 2018; Sercombe et al., 2015). These properties can be modified to increase the circulation time of NDDS following intravenous administration to allow adequate time for efficient uptake into the esophagus. They can also be modified to enhance mucosal retention and cellular uptake following topical administration in the esophagus. The effectiveness of passive targeting strategies for esophageal drug delivery has not been comprehensively investigated to date.

Active targeting approaches incorporate targeting ligands coupled to the surface of NDDS for target-specific accumulation in the target organ or diseased tissue (Hua et al., 2015, 2018; Sercombe et al., 2015). This approach has been utilized for esophageal drug delivery by exploiting disease-induced changes or organ-specific differences in the expression of receptors, adhesion molecules or proteins on the cellular surfaces of the targeted tissue. Triggered release NDDS have also been studied for esophageal drug delivery. This strategy aims to improve selective drug targeting through environmentally sensitive NDDS that react to either chemical stimuli within the target tissue (e.g. local pH, redox potential, and enzyme activity) or physical stimuli delivered from an external source (e.g. laser or light, temperature, EMF or radiation, and ultrasound) (Hua et al., 2015, 2018; Sercombe et al., 2015). Understanding the fundamental interaction of NDDS with the esophagus will help to determine the physicochemical properties and compositions important for both systemic and topical drug delivery to the esophagus.

#### Passive targeting NDDS for esophageal drug delivery

Passive targeting NDDS approaches for esophageal targeting have not been comprehensively investigated to date. There is only one research study available that is focused on liposomal irinotecan (LY01610) in patients with advanced esophageal squamous cell carcinoma (ESCC) in a Phase 1 clinical trial (Liu et al., 2021). LY01610 is a formulation of irinotecan hydrochloride encapsulated within PEGylated liposomes, which aims to protect the drug from premature conversion and activation in the liver and prolong plasma drug concentration (Liu et al., 2021). Irinotecan must be converted to its active metabolite SN-38 by carboxylesterase primarily in the liver to be clinically effective (Iyer et al., 1998). Liposomal encapsulation enables sustained release of irinotecan to lower the maximum plasma concentration (C_max_) to alleviate adverse effects and toxicity. The leaky vasculature in tumors has also been reported to enhance the accumulation of nanoparticles in the tumor tissue (Sercombe et al., 2015). Liu et al. (2021) compared the pharmacokinetics of SN-38 in their study with that from a phase 1 study evaluating conventional irinotecan at 180 mg/m^2^ (both administered via intravenous infusion) (Rothenberg et al., 1993). LY01610 showed significantly lower C_max_ of SN-38 at the maximum-tolerated dose of 90 mg/m^2^ compared to conventional irinotecan (mean: 6.45 vs 26.2 ng/mL) as well as longer half-life (39.45 vs 19.7 h) and higher AUC_inf_ (i.e. area under the concentration-time curve from time zero extrapolated to infinity) (475.81 vs 367.6 ng/mL*h). Interestingly, the AUC_inf_ of SN-38 achieved with LY01610 at 60 mg/m^2^ (455.42 ng/mL*h) was higher than of conventional irinotecan at 180 mg/m^2^, suggesting potentially improved therapeutics with lower risk for toxicity. This is the only study to date that has evaluated NDDS for esophageal drug delivery in humans. Further studies to investigate the efficacy of LY01610 for treating ESCC is warranted using established chemotherapy protocols that include irinotecan, which is primarily based on combination chemotherapy.

#### Active targeting NDDS for esophageal drug delivery

There are several studies that have evaluated active targeting approaches that exploit various esophageal specific components for drug targeting to this organ. For example, Jiang et al. (2018) developed GE11 tagged oridonin loaded graphene oxide nanoparticles (Ori-GE11-GO) for potential treatment of esophageal cancer. GE11 peptide exhibits high affinity to epithelial growth factor receptors (EGFR) (Genta et al., 2017), which are overexpressed in esophageal cancer at ∼13-fold higher compared to Barrett’s esophagus mucosa (Cronin et al., 2011) and ∼20-fold higher compared with normal esophageal mucosa (Kashyap & Abdel-Rahman, 2018). Higher EGFR expression was confirmed in this study on human esophageal cancer cells (KYSE-30 and EC109) by western blot analysis and significant cellular association of GE11-GO (average size of ∼196 nm with an average zeta potential of −41.2 mV) was demonstrated on these cancer cells (>3-fold) compared with normal human esophageal epithelial HEEC cells (Jiang et al., 2018). However, it should be noted that cellular binding and uptake were not differentiated in the methodology used for this study. GE11-GO showed minimal cytotoxicity on both KYSE-30 and EC109 cells across the concentration range up to 320 µg/mL, whereas Ori-GE11-GO (average size of 200 nm with an average zeta potential of −39.3 mV) resulted in strong concentration-dependent inhibition on both esophageal cancer cell lines (Jiang et al., 2018). For example, KYSE-30 cell viability was less than 50% for Ori-GE11-GO at concentrations greater than 80 µg/mL. In order to determine the clinical potential of this technology, comprehensive *ex vivo* and *in vivo* studies are required to evaluate pharmacokinetics, efficacy, and safety in comparison to appropriate control groups (including non-targeted nanoparticles).

CD44 targeting using hyaluronic acid has also been investigated for esophageal drug delivery. CD44 is a hyaluronic acid membrane receptor that is overexpressed in solid tumors (∼6 to 7-fold higher than in healthy tissues) (Xu et al., 2021). Xu et al. (2021) developed copper ion and disulfiram (1:2 ratio) loaded nanoparticles composed of hyaluronic acid and polyethyleneimine (NP-HPDCu^2+^) to target esophageal tumors in *in vitro* and *in vivo* studies. The average diameter of NP-HPDCu^2+^ was 330.7 nm and the zeta potential was 16.9 mV. The nanoparticles were shown to mainly distribute around the cells, likely owing to their larger particle size, which enables targeted drug release in the cancer foci. Significantly higher cytotoxicity and apoptotic activity were demonstrated with NP-HPDCu^2+^ on esophageal cancer cells (Eca109) compared to the control groups − 5-fluorouracil (5-FU) and disulfirum + copper ions (DSF/Cu^2+^). For example, cell viability at 24 hours post-treatment on Eca109 cells was approximately 18%, 38% and 90% for NP-HPDCu^2+^, DSF/Cu^2+^, and 5-FU, respectively. The corresponding *in vivo* study was performed in the Eca109 xenograft tumor model (subcutaneous injection of tumor cells into the flank region of BALB/c nude mice), in which mice were given NP-HPDCu^2+^ and DSF/Cu^2+^ by intragastric administration (for systemic delivery) and 5-FU was administered by intraperitoneal injection – all treatments were administered at 5 mg/kg once every two days for 21 days. Slower tumor growth rate (∼435 mm^3^ at day 21) was reported for NP-HPDCu^2+^ compared to free disulfiram/copper ions (∼625 mm^3^), 5-fluorouracil (∼935 mm^3^), and no treatment (∼1250 mm^3^). No significant toxicity to other organs were observed in the study (e.g. heart, liver, spleen, lungs, and kidneys) using hematoxylin and eosin (H&E) staining of the tissues. Further studies are required to determine the pharmacokinetics of NP-HPDCu^2+^ following intragastric administration, including the mechanism and degree of systemic absorption and clearance as well as real-time biodistribution following administration. This would provide important information on the clinical applicability of this nanoparticulate platform.

An additional NDDS approach that has been used to target the esophagus involves fuzing membrane vesicles extracted from esophageal cancer cells onto the surface of nanoparticles to create biomimetic nanomedicines. These systems offer several proposed advantages owing to the natural composition of the membranes, including expression of various membrane proteins, ligands, and receptors for active targeting (Zou et al., 2020). Coating nanoparticles with membrane vesicles is also suggested to allow them to avoid immune system detection, thereby increasing circulation half-life following intravenous administration (Zou et al., 2020). For example, Gao et al. (2021) developed PEGylated doxorubicin and curcumin loaded PLGA nanoparticles coated with esophageal cancer cell (TE10) membrane (PEG-TE10-PLGA@Cur+DOX) for the treatment of multidrug resistant esophageal carcinoma (average size of ∼177 nm with an average zeta potential of −17 mV). Incorporation of polyethylene glycol (PEG) is claimed to hinder opsonization and clearance of nanoparticles by the reticuloendothelial system (RES) following intravenous administration (Sercombe et al., 2015; Hua et al., 2018). Biodistribution of the nanoparticles were evaluated in BALB/c nude mice that were injected with TE10-DOX cells into the subcutaneous breast (Gao et al., 2021). When the tumor size was ∼300 mm^3^, nanoparticles labeled with a fluorescent marker (DiR) were administered intravenously as a single dose and biodistribution monitored using the IVIS system (in vivo optical imaging system) as well as fluorescence intensity measured in collected blood samples (0, 2, 24 and 48 h). It was reported that mice in the PEG-TE10-PLGA@DiR group maintained a higher blood drug concentration 48 hours post-injection (2.5-fold higher compared to PLGA@DiR and 1.7-fold higher compared to TE10-PLGA@DiR); however, it should be noted that drug concentration was not directly assessed (inferred indirectly by measuring DiR). Similarly, *in vivo* imaging of live animals showed enhanced accumulation into the xenografted tumor of mice administered PEG-TE10-PLGA@DiR group (2-fold higher compared to PLGA@DiR and 1.4-fold higher compared to TE10-PLGA@DiR). *In vivo* efficacy studies were started when the inoculated tumor cells grew to ∼100 mm^3^; treatments (5 mg/kg) were then administered every 3 days for 16 days. Results at the end of the study showed that PEG-TE10-PLGA@Cur+DOX had significantly reduced tumor volume compared to TE10-PLGA@Cur+DOX (2.7-fold), PLGA@Cur+DOX (4-fold), and control PBS group (7-fold). Correspondingly, survival studies using the same treatment regimen but extended to 40 days showed ∼93% survival of mice treated with PEG-TE10-PLGA@Cur+DOX compared to the control groups (∼30% survival in TE10-PLGA@Cur+DOX group). Unfortunately, none of the mice treated with PLGA@Cur+DOX survived beyond day 20.

Another example of a biomimetic NDDS was developed by Jun et al. (2020), whereby egg yolk lipid nanoparticles (EYLNs) were coated with leukocyte plasma membrane (mEYLNs) and loaded with a chemotherapy drug (doxorubicin) and small interfering RNA against the lipid anabolic metabolism gene (siLPCAT1). LPCAT1 has been reported to be overexpressed in esophageal cancer tissues and its interference inhibits proliferation, invasion, and metastasis of esophageal cancer cells. These nanoparticles were designed to actively target esophageal cancer cells due to the LFA-1 highly expressed leukocyte membrane coating. The average diameter of mEYLNs-Dox/siLPCAT1 was ∼136 nm and the zeta potential was −21.18 mV. Compared with the non-targeted nanoparticles (EYLNs-Dox/siLPCAT1), mEYLNs-Dox/siLPCAT1 were more easily internalized by KYSE-150 esophageal cancer cells. The difference was significant across all time points, with the highest internalization difference reported as 1.4-fold at 24 hours incubation based on fluorescence intensity. Significant inhibition of proliferation (1.7-fold), migration (2.6-fold), and metastasis (1.9-fold) of KYSE-150 esophageal cancer cells were also demonstrated with mEYLNs-Dox/siLPCAT1 in comparison to EYLNs-Dox/siLPCAT1 *in vitro*. Using a xenograft tumor model in BALB/c nude mice (subcutaneous injection of KYSE-150 cells into the flank region), treatments were administered intravenously (equivalent to 5 mg/kg of doxorubicin) every 6 days for a total of 5 times. At the end of the study (day 30), significant tumor growth suppression was reported with mEYLNs-Dox/siLPCAT1 compared to EYLNs-Dox/siLPCAT (2.6-fold), EYLNs-Dox (4.8-fold), free doxorubicin (8.2-fold), and PBS control (16.4-fold). Histological and biochemical analyses did not identify any obvious pathologic changes in other harvested organs (heart, liver, kidneys, spleen, lungs) for the mEYLNs-Dox/siLPCAT1 group.

Ligand targeted NDDS for esophageal targeting have shown initial results; however, current studies are predominantly focused on intravenous administration (or intragastric administration for systemic delivery). There is a general lack of comprehensive data on the specificity and safety of these platforms at both the cellular level and whole animal model. This includes comprehensive pharmacokinetic studies as well as mechanistic studies of degradation and clearance following *in vivo* administration. Further studies are therefore necessary to validate the value of this approach for clinical translation.

#### Triggered release NDDS for esophageal drug delivery

Triggered release NDDS take advantage of the biological differences at the site of disease (e.g. pH or redox gradients) or use external stimuli to trigger the release of encapsulated compounds from nanoparticles at specific tissues or organs in the body (Hua et al., 2015; Hua et al., 2018). For example, pH responsive NDDS can be developed by incorporating ionizable groups or polymers (e.g. amines, phosphoric acids, carboxylic acids, sulfonic acids, ammonium salt) with ionizable backbones to the nanoparticles such as polyacrylic acid, polyethyleneimine, poly(methyacrylic acid), poly(ethylacrylic acid), poly(propylacrylic acid), poly(butyl acrylate) acid, poly(N-isopropylacrylamide), and poly(glycolic) acid (Liu et al., 2014). Similarly, redox responsive NDDS can be developed by incorporating materials that are susceptible to oxidation, including poly(propylene sulfide), poly(vinylferrocene), boronic esters, thioketal, and (di)selenide groups (Phillips & Gibson, 2014).

There has only been one study to our knowledge that has explored triggered release from internal stimuli for esophageal drug delivery. Based on the characteristic acidity of the tumor microenvironment, Deng et al. (2020) developed pH-responsive docetaxel and curcumin loaded polyethyleneimine (cationic polymer) and PEGylated nanoparticles (T7-NP-DC) for esophageal cancer. The nanoparticles (average size of ∼228 nm for empty and 313 nm for drug loaded) were also tagged with T7 peptide for active targeting to tumor cells. *In vitro* studies showed that drug release from T7-NP-DC was 3.3-fold and 4.9-fold higher for docetaxel and curcumin, respectively, in pH 5.5 solution compared to PBS (pH 7.4) – equating to a cumulative release of 86.8% for docetaxel and 60.2% for curcumin at 48 hours in pH 5.5 solution. In addition, T7 peptide conjugation significantly increased *in vitro* uptake (qualitative data) of nanoparticles and apoptosis (3-fold) of KYSE150 cells (esophageal squamous cell carcinoma cell line) compared to non-targeted nanoparticles (NP-DC). Corresponding *in vivo* studies in a xenograft tumor model in BALB/c nude mice (subcutaneous injection of KYSE-150 cells into the flank region) showed that intravenous administration of T7-NP-DC every alternate day for a total of 12 days (6 injections in total) reduced the tumor volume by 3.7-fold and 7.1-fold compared to non-targeted nanoparticles (NP-DC) and no treatment (PBS control), respectively. Histological and hematopoietic analysis did not identify any obvious damage in the major organs (heart, liver, kidneys, spleen, lungs) or blood among all the nanomedicine treatment groups.

Triggered release NDDS from external stimuli have been investigated in several studies for esophageal drug delivery. NDDS with magnetic particles or photosensitizers can facilitate targeted drug release by the application of electromagnetic field or activation by light/laser sources of relevant wavelength (≥700nm), respectively. For example, Choi et al. (2021) developed magnetically guidable microparticulate drug carriers that have bioadhesive properties using a bioengineered mussel adhesive protein (MAP). These microparticles (average diameter of ∼57 µm) are embedded with magnetic iron oxide (IO) nanoparticles to enable magnetically guided targeting of the esophagus following oral administration. Magnetic mediated retention properties of the microparticles (MAPIO) were evaluated in BALB/c nude mice with a neodymium magnet adhered to the anterior cervical tissue using a plaster. Fluorescence imaging confirmed retention of the MAPIO within the esophagus after oral administration in comparison to mice without application of a magnetic field, in which the formulation was found in the intestinal region. Initial *in vitro* studies encapsulating doxorubicin within MAPIO demonstrated an encapsulation efficiency of 53.6% (0.53 µg of doxorubicin per mg of microparticles) and a sustained release pattern of ∼85% release over 22 days in PBS (pH 7.4) at 37°C. The clinical applicability of these results would need to be validated in comprehensive *in vivo* studies, particularly regarding effective drug release and tissue uptake for treatment of esophageal cancer as well as duration of retention of the microparticles to the esophageal mucosa with consumption of food and fluids.

Photosensitizers incorporated into NDDS have been explored in two studies for esophageal drug delivery (Lee et al., 2018; Xiao et al., 2019). When photosensitizers are exposed to light or lasers (e.g. near infrared), the molecules become excited and are able to transfer energy to surrounding oxygen molecules, which generates reactive oxygen species (e.g. singlet oxygen) that induce cell apoptosis, vascular damage, and inflammatory responses in the local tissue (Chatterjee et al., 2008; Lucky et al., 2015). Some nanomaterials (e.g. gold nanoparticles) can absorb near infrared at different wavelengths and convert the energy into heat, resulting in hyperthermal effects that induce cell death, whilst changing the structure of polymers to achieve triggered drug release (Huff et al., 2007). For example, Lee et al. (2018) developed doxorubicin (DOX)/Au (gold)-coated nanoturf structures as an implantable therapeutic interface for near infrared-mediated on-demand hyperthermia chemotherapy. The nanoturf structure (∼100–600 nm depth and 0.5–3 µm diameter holes) was applied on an esophageal stent to produce sustained anticancer treatment (chemotherapy and hyperthermia) to prevent tumor recurrence on the implanted surface. The highly porous nanoturf structure can be used as a drug reservoir, with the thin gold layer serving as a drug passivation film and effective light absorber. Interestingly, *in vitro* doxorubicin release profiles from the gold nanoturf structures performed in PBS (pH 7.4) only showed minor differences between with (∼25%) and without (∼18%) near infrared exposure at the 5-hour time point (10 min every hour at 808 nm and 0.3 W cm^−2^). *In vivo* studies were conducted using tissue-engineered esophageal cancer constructs that were prepared by inserting stent samples into a collagen tube embedded with OE33 cells (human esophageal adenocarcinoma cell line) and implanted subcutaneously under the dorsal skin of BALB/c nude mice. Near infrared laser (1 W cm^−2^) was applied for 5 min to the implant site of the animals and the constructs were then explanted 3 days later. There was a 5-fold increase in the percentage of apoptotic cells identified in the Au nanoturf esophageal stent group exposed to near infrared (∼25% with and ∼5% without near infrared) compared to 1.9-fold increase for the DOX/Au nanoturf esophageal stent group containing relatively small amounts of doxorubicin (1.18 μg) on the stent (∼68% with and ∼35% without near infrared). With a higher amount of doxorubicin embedded on the stent (12.07 μg), there was no difference in apoptotic index with near infrared exposure (∼96% with and ∼97% without near infrared). Although this technology is innovative, it is difficult to determine the clear value of the combined chemotherapy and hyperthermia with near infrared exposure based on the data of this study.

The second study that incorporated photosensitizers into NDDS for esophageal drug delivery was by Xiao et al. (2019), which created nanoparticles composed of albumin, Chlorin e6 (photosensitizer), and manganese dioxide (ACM-NPs) embedded into electrospun fibers that were used to cover biodegradable esophageal stents made from poly(ε-caprolactone) (PCL) and poly(p-dioxanone). The concept was for the gradual release of the ACM-NPs (average size of 63.9 nm) from the stents and accumulation into the tumor to enable effective photodynamic therapy. In addition, incorporation of manganese dioxide enables the decomposition of endogenous acidity at the tumor site and the conversion of H_2_O_2_ to produce O_2_, which alleviates the hypoxic microenvironment and enhances photodynamic therapy-mediated tumor destruction. *In vitro* results on Eca-109 cells (epithelial cell line of human esophageal carcinoma) showed significant cytotoxicity following photoactivation (660 nm light for 30 min at a power density of 5 mW cm^−2^) for both the ACM-NPs (11-fold decrease to ∼7% cell viability at 5 µm Ce6 concentration) and control albumin-Ce6 (5.4-fold decrease to ∼16% cell viability at 5 µm Ce6 concentration) electrospun fiber groups compared to the respective groups without light exposure – and this cytotoxicity was dose dependent. The efficacy of the ACM-NPs esophageal stents was evaluated in a xenograft tumor model in BALB/c nude mice (subcutaneous injection of Eca-109 cells into the dorsal region of nude BALB/c mice) and a rabbit orthotopic esophageal cancer model (VX2 tumor cells injected into the esophagus of New Zealand white rabbits) (Xiao et al., 2019). Photodynamic therapy (660 nm light at a power density of 5 mW cm^−2^ for 30 min) was only applied once in the mice studies at 24 h after stent placement, whereas it was repeated at 72 h and 120 h in rabbits (3 applications in total). The average tumor volume at day 14 was 3-fold and 24-fold lower for the mice treated with ACM-NP (∼50 mm^3^) compared to the albumin-Ce6 fibers (∼150 mm^3^) and placebo (∼1200 mm^3^) groups, respectively. In addition, ACM-NPs fibers treated mice showed a 7.5-fold reduction in relative hypoxia-positive areas in the tumors immediately after photodynamic therapy compared to those treated with albumin-Ce6 fibers (∼4% and ∼30%, respectively). Similar results were identified in the rabbit model, with the long-term survival study demonstrating a median survival time of 120 days for rabbits administered ACM-NPs stent, which is 2.6-fold and 5.3-fold longer compared to those administered albumin-Ce6 stent (45.5 days) and placebo PCL-stent (22.5 days), respectively.

Despite the limited studies available, triggered release NDDS for esophageal targeting have shown promising initial results in terms of efficacy. Most have focused on topical drug delivery following implantation of NDDS embedded into esophageal stent devices or magnetic NDDS with magnet guided localization in the esophagus. Further studies are required to comprehensively assess the safety, biodistribution, and reproducibility of these platforms based on the specific internal or external stimuli to trigger drug release. This includes the potential for systemic absorption of the NDDS or other components following detachment in the esophagus and passage through the GI tract. In addition, patient tolerability will also need to be considered for clinical translation.

## Future advances in esophageal drug delivery

Targeted drug delivery to the esophagus is a significant challenge. Owing to the physiological and pathophysiological barriers that have been addressed earlier, current pharmacological treatment for esophageal diseases generally involves off-label use of drugs in various dosage forms, including those for systemic drug delivery (e.g. oral tablets, sublingual tablets, and injections) and topical drug delivery (e.g. metered dose inhaler, viscous solution or suspension, and endoscopic injection into the esophagus). The choice of dosage form is highly dependent on factors such as the severity of the underlying condition and any pathophysiological alterations to the esophagus, which may further limit adequate drug accumulation in the esophageal tissues and require much higher doses of drugs. This can increase the risk of adverse effects and toxicity, especially in non-target organs.

Improving topical drug delivery in the esophagus has enormous potential in enhancing the way we treat patients with acute and chronic esophageal diseases, especially those requiring drugs that have low therapeutic index (i.e. margin of safety that exists between the dose of a drug that produces the therapeutic effect and the dose that produces toxicity) and/or significant adverse effects to non-targeted organs and tissues. Although there are a number of pathological conditions that affect the esophagus, the majority of the available studies for conventional formulations are focused on GERD and oesophagitis while the more innovative formulations are predominantly focused on esophageal cancer. We expect to see more studies in the future as further data are attained for these esophageal specific formulations beyond the predominantly pharmaceutical manufacturing and *in vitro* and *ex vivo* characterization assays.

Several studies that are required for clinical translation of these platforms include comprehensive efficacy, safety, and pharmacokinetics in relevant animal models of the disease. Mice and rats are often used for preclinical evaluation; however, it should be noted that their esophagus has keratinized squamous epithelium that presents an additional barrier (humans have non-keratinized epithelium) (Kararli, 1995). Despite this, the esophageal transit time is quick and governed by peristalsis, which is similar to humans despite the horizontal/vertical differences. The esophagus of pigs offers more similarities to that of humans in terms of length, actual transit time and characteristics (Kararli, 1995; Diaz Del Consuelo et al., 2005); hence, it should be considered where possible for *in vivo* evaluation. Constraints of porcine studies include limited availability of pigs compared to rodents; inexperience in handling and conducting porcine studies; inadequate facilities to house pigs for the study duration; and reduced capability for whole body analysis.

Animal models of esophageal diseases have received only minimal attention. It is clear that the pathophysiological differences of the disease in the animal model and humans should be as comparable as possible. Similarly, many esophageal cancer studies are conducted in xenograft tumor models, whereby tumor cells are injected subcutaneously into tissues outside of the esophagus (e.g. flank region of mice) (Deng et al., 2020; Gao et al., 2021; Xu et al., 2021). Although the tumors developed may be similar to human esophageal cancer, the model lacks the ability to ascertain whether the formulations are able to bypass the many biological barriers within the esophagus. In addition, preclinical studies should be conducted under appropriate randomization and blinding to minimize bias and confounding factors, as well as be evaluated against proper controls, including the current gold standard treatment(s). Such controlled conditions are currently lacking in many published studies, which makes it difficult to assess clinical applicability and translatability.

Detailed safety and toxicology assessment is essential for clinical translation of any novel formulation (Hua et al., 2018). This is particularly important for pharmaceutical dosage forms containing components that have not yet been validated for safety in humans, as is often the case here with those designed for esophagus-related diseases. In addition to *in vitro* cellular studies, specialized toxicology studies in animal models need to be used to assess short-term and long-term toxicity. Biodistribution evaluation can also predict potential toxicological responses by determining factors such as off-target accumulation in healthy tissues as well as clearance mechanisms. Implementation of real-time imaging techniques (e.g. IVIS, MRI, CT) can allow improved understanding of the degree of interaction of esophageal targeting formulations with target and non-target organs and tissues after *in vivo* administration in longitudinal studies (Arms et al., 2018).

Lastly, formulations should be assessed for their potential translatability from a large-scale manufacturing and quality control perspective (Hua et al., 2018). This includes cost-effectiveness and risk-benefit analysis compared to existing therapies, including any ‘gold standard’ treatments (where available). Patient acceptability of the formulation as well as ease of administration should also be considered. An essential requirement for clinical translation is to have access to a preparation method that allows the production of large scalable quantities of the formulation, which is also consistently manufactured at the same high level of quality and batch-to-batch reproducibility set by specifications. Therefore, platforms that require complex and/or laborious synthesis procedures generally have limited clinical translation potential. Such complex platforms, including a number of the innovative pharmaceutical approaches for topical drug delivery in the esophagus, will also need to substantiate the necessity of their composition and/or design on the clinical impact – that is, to determine if each added complexity makes a substantial difference to the clinical outcome.

## Conclusion

Although diseases affecting the esophagus are common, drug delivery to this organ is challenging. Understanding the physiological and pathophysiological barriers is important for developing successful formulations for topical esophageal drug delivery. A variety of conventional and innovative pharmaceutical strategies have been investigated for local drug delivery in the esophagus, with each having its own advantages and limitations in overcoming the biological barriers of this organ. Further research in esophageal drug formulation design and evaluation are mandatory to advance this specialized drug delivery field. Though progress has been slow and is expected to remain slow due to the formidable biological obstacles of this organ, persistency in keeping pharmaceutical innovation at the forefront of esophageal drug delivery research will undoubtedly pay-off and lead to patient benefit.
